# Darwinian properties and their trade-offs in autocatalytic RNA reaction networks

**DOI:** 10.1038/s41467-021-21000-1

**Published:** 2021-02-08

**Authors:** Sandeep Ameta, Simon Arsène, Sophie Foulon, Baptiste Saudemont, Bryce E. Clifton, Andrew D. Griffiths, Philippe Nghe

**Affiliations:** 1 Laboratoire de Biochimie, ESPCI Paris, Université PSL, CNRS UMR 8231, 10 Rue Vauquelin, Paris, France; 2 School of Chemistry and Biochemistry, Georgia Institute of Technology, Atlanta, GA USA; 3 Present Address: Simons Centre for the Study of Living Machines, National Centre for Biological Sciences (TIFR), Bangalore, India

**Keywords:** Origin of life, Ribozymes, Evolvability

## Abstract

Discovering autocatalytic chemistries that can evolve is a major goal in systems chemistry and a critical step towards understanding the origin of life. Autocatalytic networks have been discovered in various chemistries, but we lack a general understanding of how network topology controls the Darwinian properties of variation, differential reproduction, and heredity, which are mediated by the chemical composition. Using barcoded sequencing and droplet microfluidics, we establish a landscape of thousands of networks of RNAs that catalyze their own formation from fragments, and derive relationships between network topology and chemical composition. We find that strong variations arise from catalytic innovations perturbing weakly connected networks, and that growth increases with global connectivity. These rules imply trade-offs between reproduction and variation, and between compositional persistence and variation along trajectories of network complexification. Overall, connectivity in reaction networks provides a lever to balance variation (to explore chemical states) with reproduction and heredity (persistence being necessary for selection to act), as required for chemical evolution.

## Abstract (web summary)

Autocatalytic networks may have started evolution during the origin of life. Here, the authors establish a landscape of thousands of RNA networks by barcoded sequencing and microfluidics, and derive relationships between topology and Darwinian properties such as variation and differential reproduction.

## Introduction

Autocatalytic reaction networks, where chemicals collectively catalyze the synthesis of each other, are a crucial ingredient for the emergence of evolution, as they enable the maintenance and reproduction of out-of-equilibrium chemical states. Experimentally, autocatalytic chemistries built from a broad range of molecules, including organic^1^ and inorganic molecules^2,3^, macrocycles^4^, peptides^5^, DNAs^6^, and RNAs^7,8^, have been described. Theoretically, it has been shown that autocatalytic reaction networks can emerge from random pools^9–12^ and several scenarios have been proposed for how they could have supported early modes of evolution^10,13–17^. Although the proposed dynamics and suggested chemical embodiments differ, these scenarios have in common the possibility to form a diversity of autocatalytic systems, with evolution arising from transitions between such systems. Consistently, recent experiments indicate that RNA replicases (RNA-dependent RNA-polymerase ribozymes) may have emerged as components of such networks^18^, and hypercycle models propose that early replicases may have participated to networks^19^. The possibility for autocatalytic networks to evolve is significant as the spontaneous appearance of an RNA replicase with sufficient processivity to allow self-replication and enough fidelity to avoid an error catastrophe^20,21^ seems unlikely, given the length (>165 nt) and structural complexity of known replicases^22–24^.

Nevertheless, sustaining evolution in reaction networks without template-based replication is by no means trivial, and autocatalytic networks that could support the Darwinian properties of variation, differential reproduction, and heredity are not known^15,25^. Indeed, these properties need to be mediated by chemical compositions (the chemical species present and their concentrations)^26^ without the copying of a sequence. Furthermore, evolution in reaction networks may be constrained by trade-offs between these properties. For instance, robustness to environmental perturbations and persistence of compositions are necessary for selection to act, but must be balanced with variation to explore novel states.

Here, we develop a method to generate a wide diversity of reaction networks and measure the relationship between network topology and product formation in a prebiotically relevant experimental model of autocatalytic sets of RNAs^7,11,16^ (note that the resulting reaction networks are not hypercycles, which rely on template-based replication^27^). The large-scale combinatorial screen establishes a landscape of networks from which we analyzed the growth (product accumulation or reaction yield) and fraction of catalytic species (composition) as a function of network topology. We also compare the composition of networks that differ by a single catalytic species as way to assess the susceptibility of networks to the appearance of novel catalytic species. From this, we deduce network parameters that control properties central to Darwinian evolution: reproduction, interpreted as the accumulation of autocatalytic species, and variation, interpreted as changes in species fractions. We found how these parameters depend on the specificity of the interactions between the catalysts produced during the reaction and their substrates. In turn, these molecular rules are found to impose trade-offs between the Darwinian properties they control, thus predicting how evolutionary trajectories can be constrained in a scenario where networks expand by successive accretion of novel species.

**Fig. 1:**
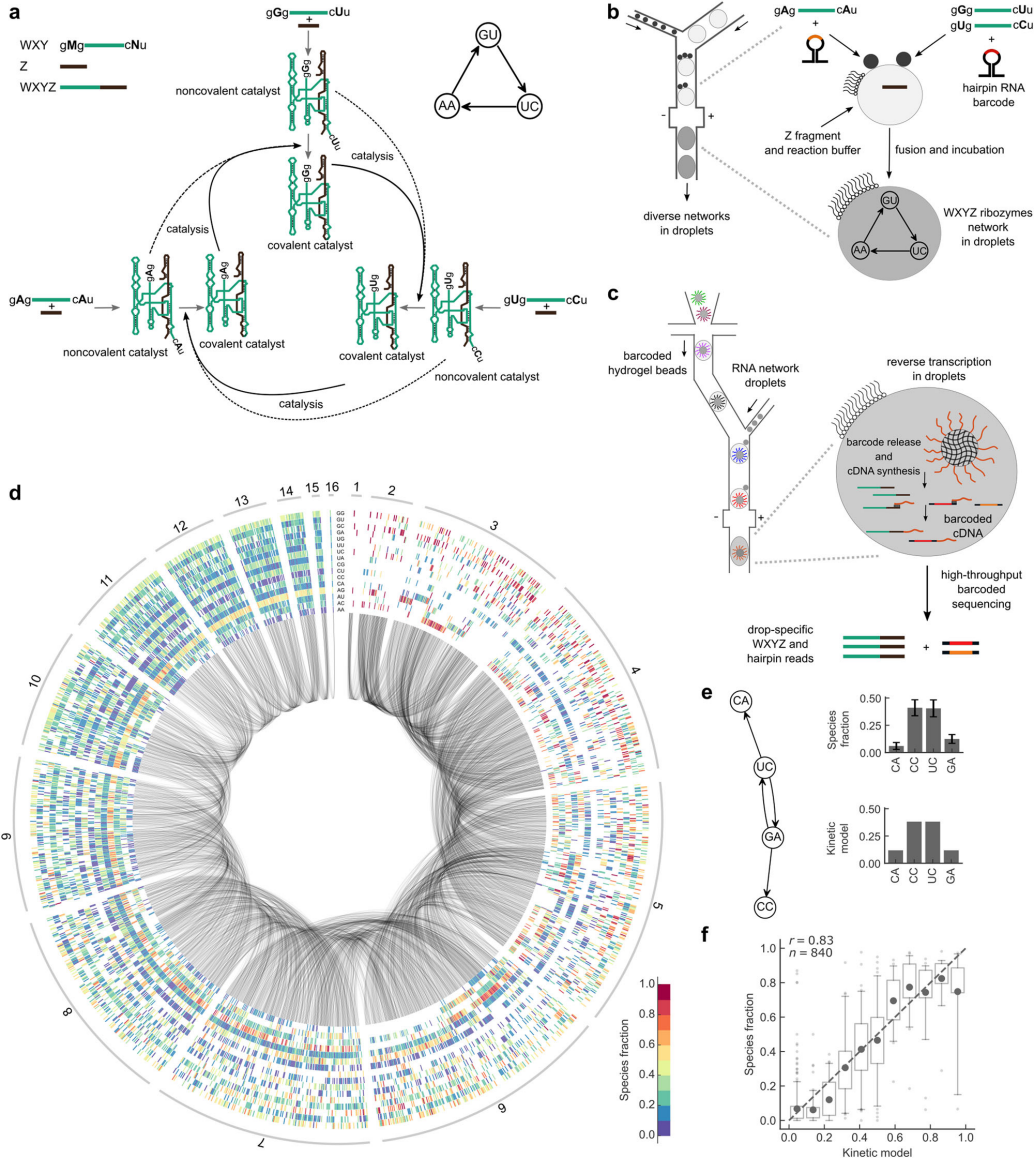
Experimental setup and RNA network compositional landscape. **a** Top left: WXY and Z fragments assemble into noncovalent and covalent WXYZ ribozymes. WXY fragments comprise IGS and tag sequences, respectively denoted gMg and cNu. M, N variations result in 16 different combinations^30^. Bottom center: M–N base-pairing determines ribozyme specificity. Here, three ribozymes form an autocatalytic cycle. Dotted and bold arrows show catalysis by noncovalent and covalent ribozymes, respectively. For clarity, only Watson-Crick IGS-tag interactions are shown (non-Watson pair are weaker but non-zero, Supplementary Fig. 6c). Top right: simplified representation showing catalytic species as nodes with their MN identity and catalytic interactions as directed edges (corresponding to dotted and bold black arrows in the bottom center panel). **b** Random sets of ~1–5 droplets (small black disks) containing different WXY mixtures are fused by electrocoalescence in a microfluidic device with a droplet containing Z fragments and the reaction buffer (larger gray disks), generating diverse reaction networks (Methods). Fragments are initially co-diluted with nonreactive hairpin RNA reporters to retrieve initial compositions by sequencing (Supplementary Fig. 1). **c** Droplet-level barcoded sequencing: after incubation, droplets are fused to another set of droplets containing DNA barcoded hydrogel beads. Barcodes are specific to each bead and prime RNA reverse transcription. **d** Compositional landscape of 1837 unique networks^48^: each ray comprises 16 boxes for the 16 MN species. Network sizes are indicated on the outer circle. A blank box indicates an absence of MN fragments. A colored box indicates the measured fraction of the MN ribozyme. Gray arcs connect networks that differ by a single substrate species. **e** Left: schematic of a network. Right: top, measured fractions of catalytic species (WXYZ concentration divided by the total concentration, data are represented as mean ± s.d. over *n* = 425 droplets); bottom, model prediction. **f** Kinetic model versus measured fractions for every ribozyme in 4-species network (*n* = 840, bins with <10 points are discarded); dark gray dots: mean values; quartile boxplots; 5–95th percentiles whiskers with flier points; dotted grey line: identity; Pearson correlation coefficient is reported (*P*-value < 1e-5).

We use a model of autocatalytic RNAs derived from the group I intron^28^ of the *Azoarcus* bacterium, where fragments, denoted WXY and Z, assemble into noncovalent complexes that catalyze the formation of more efficient covalent ribozymes^7,29^ (denoted WXYZ, Fig. 1a), which in turn catalyze, with higher efficiency, the formation of further covalent ribozymes^29^. Three nucleotide long sequences, called internal guide (IGS) and target (tag), located at extremities of the WXY fragments^29^ (Fig. 1a, top left), determine catalytic specificity by base-pairing between a ribozyme IGS and a fragment tag, combinations of which yield reaction networks of diverse connectivity (Fig. 1a, bottom center)^30^. Diversity in connectivity had been shown to lead to qualitatively distinct dynamics based on a few examples^31–33^, which we considerably expand here. In a coarse-grained representation of these networks by directed graphs (Fig. 1a, top right), a node represents the covalent ribozyme species with a given IGS and tag, and a directed edge points from an upstream ribozyme species to the downstream ribozyme species whose formation it catalyzes.

## Results

### High-throughput measurement of RNA reaction networks

We developed a method to generate and measure a high diversity of such RNA reaction networks, using droplet microfluidics coupled to barcoded Next-Generation Sequencing (Fig. 1b, c and Supplementary Fig. 1). It consists of first producing a library of 5 pL droplets containing 24 different combinations of the 16 WXY fragments (Supplementary Table 1), together with hairpin RNA reporters that do not react, but enable later identification of the mixture of WXY fragments by sequencing. The 24 initial combinations were selected among a random sample by minimizing their mutual overlap while covering as evenly as possible network sizes from 1 to 16 catalytic species after combinatorial droplet fusion (Methods). Experimentally, random sets comprising 1–5 droplets of this initial library were then electrocoalesced^34^ with a 50 pL droplet containing the reaction buffer (see Methods) and Z fragments (Fig. 1b). After incubation at 48 °C for 1 h, the composition of each droplet was analyzed by droplet-level RNA sequencing adapted from single-cell transcriptomics^35^. Hydrogel beads carrying cDNA primers with a barcode specific to each bead (Supplementary Fig. 2) were encapsulated one-by-one in droplets, which were then fused with single RNA containing droplets, the primers were released and cDNA synthesis was performed in the droplet (Fig. 1c, Supplementary Fig. 1, and Supplementary Methods). Barcoded cDNAs were recovered and sequenced. Reads from the same droplet carry the same barcode (Supplementary Fig. 3a, b).

The initial combinations of fragments (encoded by hairpin RNA reporters, Supplementary Figs. 3, 4), and the final fraction of covalent ribozymes produced during incubation were determined in 20,038 droplets, comprising 1837 unique networks, with on average 11 replicates each (Fig. 1d and Supplementary Fig. 5a), indicating a mean precision of ~6% in species fractions (Supplementary Fig. 5b). Repeatability was tested with a full experimental replicate (r = 0.84, *p* < 10^−3^ between species fractions; Supplementary Fig. 5c). Furthermore, controls with mixtures of droplets containing defined sets of ribozymes in known proportions showed a high correlation (r = 0.91, *p* < 10^−3^) between measured and expected species concentrations (Supplementary Fig. 5d–h).

We observed heterogeneous patterns of catalyst accumulation, demonstrating the existence of interdependences between reaction network components (Fig. 1d). Indeed, if catalyst were reacting independently, the relative rank between any pair of them would be conserved across all networks. On the contrary, we observed that for 63% of catalyst pairs, the ranking between the fractions of these catalysts within networks differed in at least 10% of the networks (Supplementary Fig. 6a), extending previous findings of nonconserved ranking in a small set of such networks^32^. The diversity in catalyst fractions in networks was well predicted by a kinetic model without fitting parameters (Methods) by adding the first order effective catalytic rates of noncovalent and covalent ribozymes measured independently for individual IGS-tag pairs (r = 0.83, *p* < 10^−3^, Fig. 1e, f and Supplementary Fig. 6b–d). Note that the model uses the approximations of early reaction times and weak consumption of noncovalent complexes (Methods).

### Relationship between growth, variation, and connectivity

**Fig. 2:**
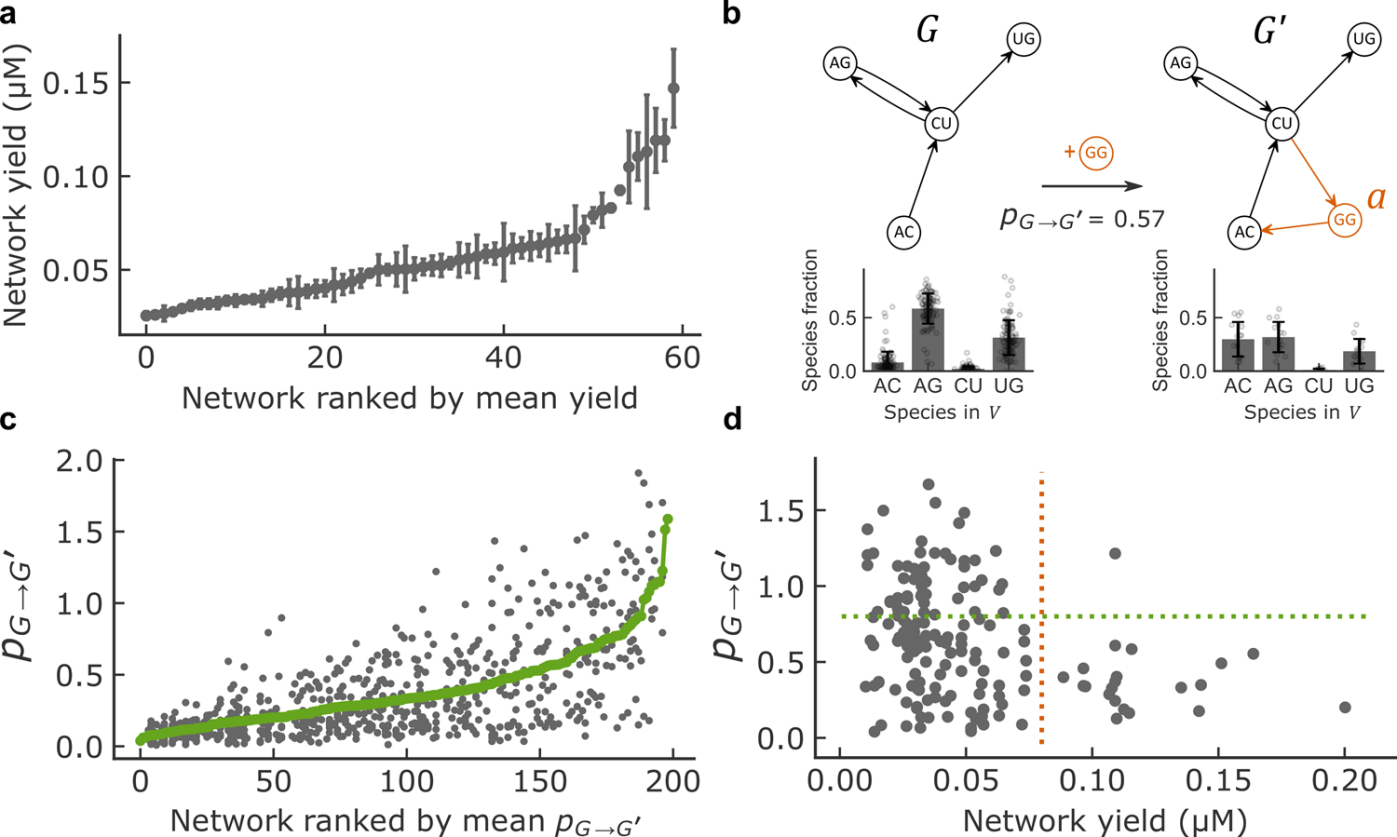
Network yield and perturbation. **a** Network total yield (total concentration of WXYZ ribozymes in µM) distribution of networks with four catalytic species (among the 1820 theoretically possible) that comprise at least 10 replicates. Data on all network sizes is provided in Supplementary Table 2. Networks are ranked from lowest to highest yield (*x*-axis) based on the mean across network replicates of the total WXYZ concentration (*y*-axis). The error bars show the Standard Error of the Mean. See Methods for further details on network yield determination. **b** Example showing the addition of catalyst *a* (GG, orange) to network *G* resulting in network *G*′. Top: schematic. Bottom: experimentally determined species fractions for the same network (data are represented as mean values ±1 s.d. over *n* = 109 droplets (*G*) and *n* = 15 droplets (*G*′). **c** Perturbation distribution of networks with four species. Networks are ranked (*x*-axis) based on the mean perturbation (*y*-axis). Each position on the *x*-axis corresponds to a certain network *G*, for which the perturbation has been computed for all possible single species additions present in the dataset, leading to several *y*-axis values corresponding to different *G*′ networks. The green line is the average perturbation taken overall *G*′ networks for each network *G*. **d** Perturbation *p*_*G*_→_*G*′_ for networks with 3, 4, and 5 species plotted against network yield in µM for perturbations involving the addition of a novel catalysts (with G/C as IGS) with at least one target. The number of strongly perturbable (*p*_*G*_→_*G*′_ > 0.8, above the green dotted line) and high-yield networks (>0.08 µM, right hand side of red dotted line) is very low compared to the null hypothesis of independence between the two properties (one-sided Fisher exact test, *N* = 162, odds ratio <0.1, 95% confidence interval 0.01–0.66, *p*-value = 1.6 × 10^−3^).

Comparing networks generated from distinct substrate sets also revealed differences in growth, quantified as the concentration of catalyst accumulated during the reaction, measured relatively to hairpin reporters of known concentration (Fig. 2a): yield varied 10-fold across networks comprising between 2 and 15 catalytic species (see Supplementary Table 2), and up to 6-fold within networks of the same size but with different topologies (Fig. 2a). The kinetics, yield and catalyst fractions of selected low-yield, mid-yield, and high-yield four species networks, identified from Fig. 2a, were also measured in bulk and found to correlate well with measurements in droplets (Supplementary Fig. 7).

We then used the landscape of networks to study compositional variation in response to the appearance of novel catalytic species. In a prebiotic system, the latter may appear stochastically as the products of rare reactions (intrinsic cause of variation), or due to environmental changes in physico-chemical conditions (extrinsic cause of variation)^36^, including access to novel chemical reactants^37,38^. To study possible outcomes of such scenarios, we compared reaction conditions that differ in a single reactant species allowing the formation of a novel catalytic species. We interpreted the change in species fractions induced by the extra species as a perturbation of the network, in the sense that the system with and without the extra species have different fractions of the common species. Note, however, that perturbation of a preformed network by addition of a new species may not result in the same species fractions: if the final reaction network has multiple stable states, its state may depend on the composition before adding the novel species.

More specifically, we compared networks *G* and *G*′ differing by a single catalytic species denoted *a* (networks connected by a curved line in Fig. 1d). Denoting *V* the set of species common to *G* and *G*′, we define the network perturbation as *p*_*G*_→_*G*′_ = $\mathop {\sum}\nolimits_{v \in V} {\left| {y_v^\prime - y_v} \right|}$ where *y*_*v*_ and $y_v^\prime$ are the respective fractions of species *v* in *G* and *G*′, both normalized within *V* (Fig. 2b). By definition, the maximum is $p_{G \to G{\prime}} = 2$ and is reached for a full switch in species composition, for example going from (*y*_*u*_, *y*_*v*_) = (0, 1) to $(y_u^\prime ,y_v^\prime ) = (1,0)$ in $V = \{ u,v\}$. Averaging for each network the effect of all perturbations (green line, Fig. 2c) revealed large differences from networks that are marginally (mean$\bar p_{G \to G^{\prime}} = 0.1$) to strongly ($\bar p_{G \to G} = 1.5$) susceptible to species addition. Furthermore, we observed that high-yield networks tend to display small perturbation values, implying a trade-off between growth and variation (Fig. 2d, Supplementary Fig. 6e).

We next aimed to interpret the diversity of observed responses based on network connectivity. The kinetic model does not provide such a direct interpretation as it consists of a mixture between two regimes, where either only noncovalent or only covalent ribozymes are active. For noncovalent ribozymes only, product fractions should be predicted by in-degree centrality, a network-theoretic measure which accounts only for catalysis by directly upstream catalysts^33^. In contrast, for covalent ribozymes only, product fractions should be predicted by eigenvector centrality, which accounts for longer catalytic chains^33^. Despite these differences, the in-degree centrality was found to be a good approximation of the eigenvector centrality for our dataset (R = 0.83, *p*-value < 10^−5^, Supplementary Fig. 8a), as is already known in general^39^, except for species strongly catalyzing their own production, whose fraction is underestimated (13% of the dataset, Supplementary Fig. 8b).

The in-degree centrality approximation allowed an analytical derivation of the total perturbation (Supplementary Note):

1 $$p_{G \to G^{\prime}} = 2n\frac{{1 - n\frac{m}{{\sigma _G/e}}}}{{\sigma _G/e + n}}$$

**Fig. 3:**
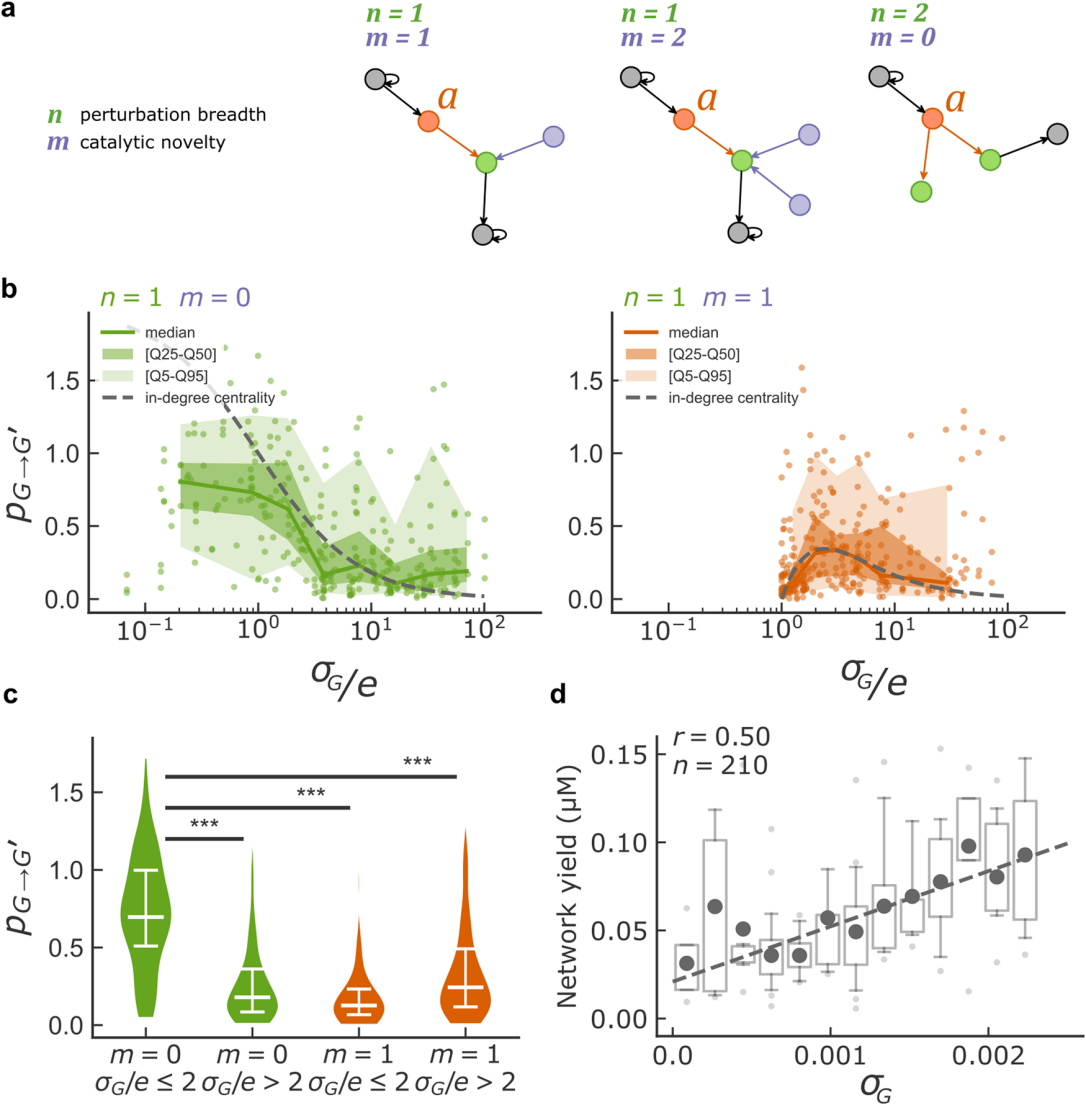
Network perturbation analysis. **a** Schematic of three situations where the parameters *n*, the number of targets of added catalyst *a*, and *m*, the number of catalysts sharing the same IGS as *a*, have different values. **b** The perturbation $p_{G \to G^{\prime}}$ is plotted against *σ*_*G*_/*e* for different values of the parameters *m* and *n* for the networks with 3 and 4 species before addition of the new catalyst *a*. Dots are the measurement for one perturbation, the colored line is the median, shadings indicate 25–75 and 5–95 percentiles, and the dotted grey line is the in-degree centrality prediction. To compute percentile statistics, data points were binned in 10 bins along the x-axis and bins with fewer than 10 points were discarded. **c** Violin plot (with the median and interquartile range in white) of $p_{G \to G^{\prime}}$ with catalytic innovations (*m* = 0, green, *n* = 101 and 202 perturbations) or without catalytic innovations (*m* = 1, orange, *n* = 130 and 166 perturbations) at high and low values of *σ*_*G*_/*e*. Two-sided Mann–Whitney U test p-values are reported (***p-value < 0.001, *p* = [8e-21, 2e-22, 2e-15] from left to right, H0: equal means). **d** Network yield (µM, see Methods, *n* = 210 networks) is plotted against *σ*_*G*_ for network with four species. Dark gray dots are mean values, quartile boxplots have 5–95th percentiles whiskers with flier points. The dotted gray line is the identity line. Pearson correlation coefficient is reported (*p*-value < 0.001).

The predicted perturbation depends on four parameters, which characterize the initial network and the added catalytic species (Fig. 3a): (i) *n*, which we call perturbation breadth, is the number of catalytic species whose formation is catalyzed by the new catalyst *a* added to the network; (ii) *m*, which we call catalytic novelty, is the number of species already present in *G* that catalyze the same reactions as the newly added catalytic species *a* (i.e., in our case ribozymes already present with the same IGS as *a*, novelty being higher for lower *m*); (iii) *σ*_*G*_, which we call the background strength, is the sum of all catalytic rates of the catalytic species of the initial reaction network, and; (iv) *e* is the catalytic rate of the reactions catalyzed by catalyst *a* (as determined by the ribozyme IGS).

The influence of parameters *σ*_*G*_, *e*, *m*, and *n* on $p_{G \to G^{\prime}}$ predicted by the in-degree approximation (Eq. 1) and without fitting parameter (using the catalytic rates measured independently, Supplementary Fig. 6) were well verified experimentally for network perturbations (Fig. 3b). For catalytic innovations (*m* = 0, Fig. 3b left), $p_{G \to G^{\prime}}$ decreased with the normalized background strength *σ*_*G*_/*e* (the sum of catalytic rates of all catalysts already present in the network divided by the rate of the reactions catalyzed by the new catalyst). When a catalyst with the same specificity (same ribozyme IGS) as the added catalyst was already present in the network (*m* = 1, Fig. 3b right), perturbations were also low for large background strength *σ*_*G*_/*e*, but peaked at intermediate values. However, perturbations were smaller than in the case *m* = 0 over the whole range of background strength. Theory confirmed that for other values of *m* and *n* large perturbations are observed only for *m* = 0 (Supplementary Fig. 9). Overall, the largest total perturbations required a catalytic innovation (*m* = 0) to be combined with a low normalized background strength (*σ*_*G*_/*e* ≤ 1) (Fig. 3c). Similarly, predictions for perturbations of single species fractions were verified experimentally (Supplementary Fig. 10).

Consistently, the attenuation of perturbations with large *σ*_*G*_/*e* combined with the weak but significant correlation between growth and *σ*_*G*_ (r = 0.5, *p* < 10^−5^, Fig. 3d) explains part of the trade-off between yield and perturbation reported in Fig. 2d.

### Interplay between robustness and variation

**Fig. 4:**
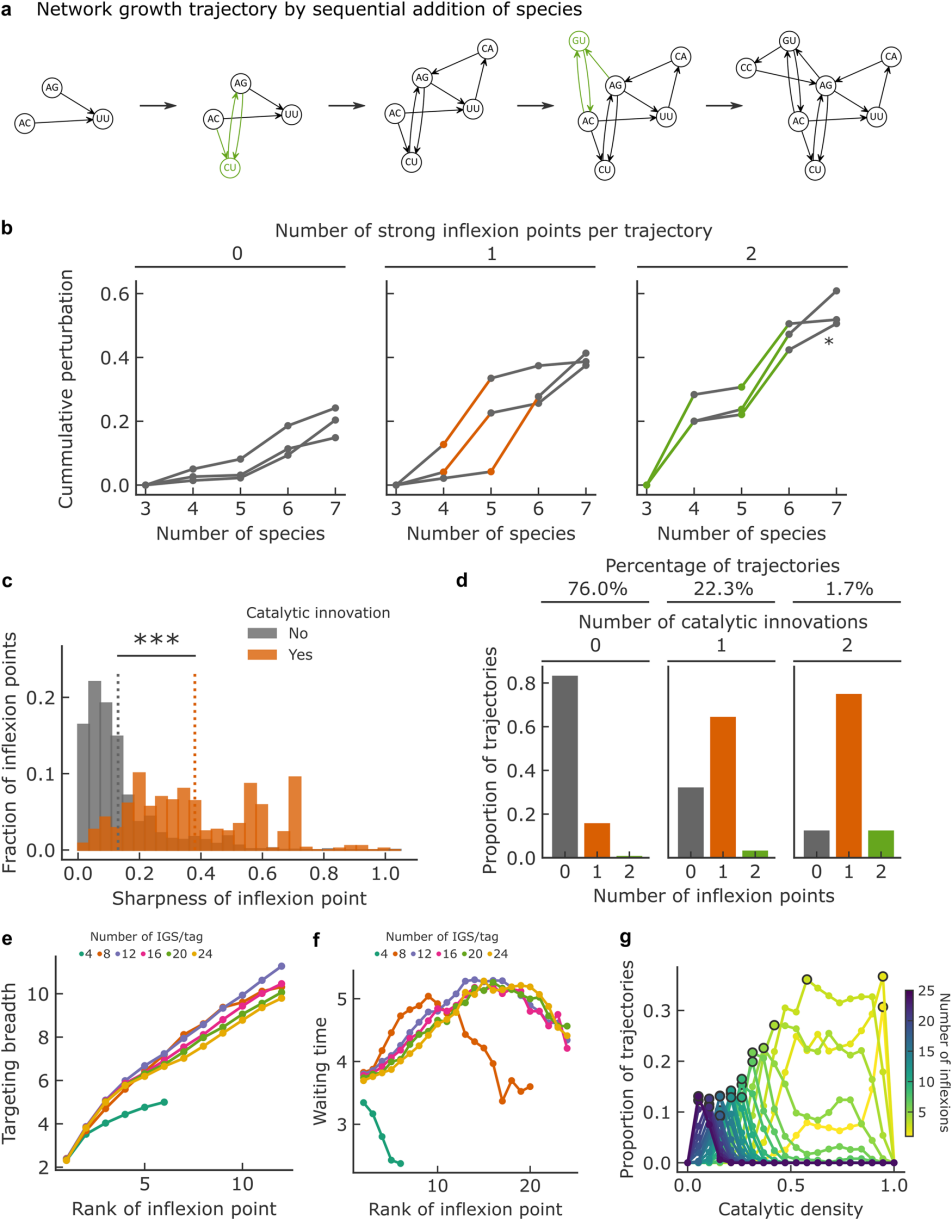
Perturbation dynamics across trajectories of network growth by species addition. **a** Example of a network growth trajectory where at each step, a new catalyst is added. Added catalysts resulting in strong perturbations (see panel **b**) are in green, and correspond to the IGS/tag pairs C–G and G–C. **b** Examples of measured cumulative perturbation over trajectories, plotted against the number of species additions, by number of strong inflexions points (colored). The latter are determined as the top 25% in sharpness (absolute value of the third derivative, Supplementary Fig. 11a) measured overall trajectories. The asterisk (*) denotes the perturbation trajectory of the example shown in panel **a**. **c** Distribution of sharpness for inflexion points associated with a catalytic innovation (orange, *n* = 4,462) or not (gray, *n* = 934). Catalytic innovations are defined as the introduction of strong IGS/tag interactions (CG or GC, Supplementary Fig. 6b, c) that were not present until node addition. The dotted line is the mean of each distribution and the significance of the difference between the two distributions is reported (Two-sided Mann–Whitney U test, p-value < 1e-5, H0: equal mean). **d** Distribution of the number of inflexion points within the 75th percentile sharpness per trajectory, depending on the number catalytic innovations per trajectory. **e**–**g** Computational study of network growth trajectories for chemistries with varying number of specific interactions (IGS/tag pairs) and varying degrees of catalytic density. Inflexion points are determined, as before, based on their sharpness (within the 75th percentile) here computed along a random sample of 1000 trajectories growing from 2 to 100 nodes. Catalytic density (probability of one species to catalyze the formation of another) is varied by random removal among the pool of all possible specific IGS/tag interactions. **e** Targeting breath (number of targets) of catalysts causing strong perturbations, as the function of the inflexion rank. **f** Waiting time (number of species additions) between two strong inflexions, as a function of the inflexion rank (e.g.: rank 5 is the fifth inflexion observed along a growth trajectory). **g** Proportions of trajectories with a given number of strong inflexion points plotted against catalytic density for a chemistry comprising up to 24 different IGS/tag pairs.

To test the interplay between robustness and variation in scenarios where novel catalytic species would appear either spontaneously^15^ or due to changes in reactants provided from the prebiotic milieu^36,40^, we analyzed cumulative perturbations across trajectories of network growth, starting from networks with three catalytic species, then randomly adding one catalytic species at a time (Fig. 4a). We have seen that strong perturbations require catalytic innovations (*m* = 0), Eq. 1 then reducing to $p_{G \to G^{\prime}}$ = 2/(1 + *σ*_*G*_/*ne*). The ratio *σ*_*G*_/*ne* expresses a second trade-off, between the robustness induced by the background strength of the network *σ*_*G*_, and the variation induced by the novel species as characterized by *n* and *e*.

As for a large perturbation to occur, *ne* must be comparable to the *σ*_*G*_ of the perturbed network, *σ*_*G*_ would roughly double after a strong perturbation, enhancing robustness to further variation. Consequently, strong variations should be followed by small ones, and result in inflexions (change in curvature) in cumulative perturbation trajectories. We quantified the corresponding inflexions by their sharpness (third derivative at inflexion, Supplementary Fig. 11a), and categorized them as strong when in the top 25% of sharpness (Fig. 4b). Comparing the distributions of sharpness for all inflexion points showed that they are, as predicted, significantly sharper for catalytic innovations (Fig. 4c). Consistently, the number of strong inflexions correlates with the number of catalytic innovations per trajectory (Fig. 4d). Note that the presence of strong perturbations, thus of inflexions, can depend on the order of species appearance, as seen when comparing alternative trajectories starting and ending at the same networks (Supplementary Fig. 12).

Although introducing a catalytic species causing a strong perturbation buffers the resulting network against further perturbations, subsequent variations along trajectories are still possible, as exemplified in Fig. 4a and b. By computationally analyzing trajectories for an extended repertoire of specific interactions, we found that sustained variation requires the addition of species of increasing targeting breadth *n* (Fig. 4e) and increased waiting time between variation events (until the diversity of specific interactions, such as IGS/tag pairs, saturates) (Fig. 4f). The former allows the even larger *σ*_*G*_ values to be overcome, while the latter corresponds to the buildup of weakly connected nodes that can become targets.

Additionally, the trade-off between *σ*_*G*_ and *ne* translates at the level of trajectories. Indeed, ‘sparse’ chemistries (with a low probability for a given species to catalyze the formation of another catalytic species) are prone to few but strong variations (sudden changes in composition), whereas ‘dense’ chemistries (with a high probability for a given species to catalyze the formation of another catalytic species) are prone to many but weak variations (gradual changes in composition) (Fig. 4g, Supplementary Fig. 11b).

## Discussion

A first general constraint to achieve evolution in chemical networks resulted from theoretical studies, which established that the density of catalytic interactions must exceed a threshold for network self-reproduction^11^. Our experimental results show that connectivity in reaction networks further poses conditions on the ability for networks to evolve, as network topology modulates both growth and variation in response to the appearance of novel species. Indeed, in autocatalytic networks, Darwinian evolution is in principle possible despite the absence of template-based replication, but relies on the appearance (due to rare reactions or environmental changes) of catalytic species that are sustained by autocatalysis and modulate the composition of a pre-existing network^10,15^. This experiment shows that a diverse growth and variation dynamics could be generated from combinations of only a few specific interactions, raising the possibility to synthesize chemistries with the capacity to evolve based on reasonably sized spaces of chemical interactions.

What then, are the requirements for reaction networks to evolve? First, we found that network-level structures constrain properties that emerge at the level of chemical compositions. Specifically, network topology imposes trade-offs between growth (favored by higher connectivity) and variation (favored by lower connectivity), and between variation (favored by lower connectivity) and robustness to environmental changes (favored by higher connectivity). These considerations are directly relevant to scenarios where early evolution is driven by environmental heterogeneity^36,40^. Indeed, in prebiotic scenarios such as Dynamical Kinetic Stability^41^, evolution depends on a balance between persistence of chemical compositions, thus robustness to environmental changes, and exploration of novel compositions, thus susceptibility to perturbations. These evolutionary trade-offs and the connectivity rules imply a ‘Goldilocks’ range (neither too high not too low) in the density of catalytic interactions for evolution to be possible.

Second, the observed dynamics points to the nonobvious role of self-assembly. Self-assembly has been demonstrated to robustly drive self-reproduction in other systems, as for example in tubular assemblies of molecules^4^. In our case, catalysis from self-assembled molecules (noncovalent ribozymes) enables the onset of self-reproduction, but leads to relaxation of chemical compositions toward states determined by the substrates, rather than by the covalent catalysts transmitted from pre-existing networks (Supplementary Fig. 13). This limits the potential for a diversity of heritable states in our experimental model of autocatalytic sets. Indeed, such a diversity depends on the ability of different sets of catalysts to lead to reproduction of their own types in the same proportions over time. Reducing catalysis by self-assembled complexes consequently appears to be a critical factor to obtain multiple states with sufficient heredity to evolve.

Ultimately, provided that connectivity is balanced, and heredity mechanisms are strong enough, evolution could occur in autocatalytic sets if the system is maintained out-of-equilibrium during cycles of compartmentalization and selection^42,43^. In these conditions, the stochastic appearance of novel catalysts, either due to rare reaction or environmental inputs, could in turn allow the appearance of novel self-sustaining autocatalytic networks^15^. Dilution and selection may then lead to the disappearance of other catalysts^44^, providing various modes of heritable variation in autocatalytic sets. Such autocatalytic networks dynamics may provide a gradual route by which novel chemical states can be explored and selected in a Darwinian manner^15,45,46^. The next step would then be to understand how Darwinian evolution in networks can select chemistries of increasing complexity, which may have been favored by environmental cycles of temperature, salinity or other physico-chemical conditions^18,47^. A major challenge is to understand how such network evolution may have led to evolution of template-based replication, as observed in living systems today.

## Methods

### Materials

All experiments used DNAse/RNase free water (Thermo Fisher Scientific Product No.: 10977035). Chemicals were purchased from Sigma–Aldrich unless specified otherwise. 4-(2-Hydroxyethyl)-1-piperazinepropanesulfonic acid (EPPS) was purchased from Alfa Aesar (Product no.: J60511, CAS no.: 16052-06-5). Denaturing polyacrylamide gels (12%) were prepared using gel stock solution (Roth, Product no.: A431.1) and run in 1X Tris-Borate EDTA buffer (TBE). DNA/RNA oligonucleotides (Supplementary Data 1) were obtained from IDT DNA technologies.

### Software packages

Sequencing data processing and analysis was performed using custom software written in Python 2.7.16. The compositional landscape (Fig. 1d) was prepared used ‘Circos’ software^48^ (Version 0.69-6).

### Experimental methods

#### Transcription of RNAs

RNAs were in vitro transcribed as described in ref. ^31^. In the dsDNA template for the WXY fragments the IGS is duplicated at position 25 to allow identification by sequencing. This is done by site-directed mutagenesis and producing plasmids bearing respective IGS duplicated at 25th position (see ‘Duplication of IGS’ Supplementary Methods). For PCRs, plasmids (at 25 pg/µL) were mixed with 0.5 µM of respective primers (Oligo 1–4 and 6–9, Supplementary Data 1) as forward and reverse primers in 1× PCR buffer (Thermo Scientific), 0.2 mM dNTPs, 0.01 U/µL of polymerase (Thermo Scientific Phusion Hot Start II, Product No.: F459) using the following protocol: initial denaturation 98 °C/30 sec, then 25 cycles of denaturing 98 °C/10 sec, annealing and extension 57 °C/1 min, and a final extension of 72 °C/5 min. PCR products were ethanol precipitated by adding 1/10th volume of 3 M Na-Ac and 1.2 volume of 100% ethanol to the PCR reaction and centrifuging at 13.6 × *g* for 60 min at 4 °C. Pellets were vacuum dried, resuspended in 20 µL of water and used for in vitro transcription reactions as described in ref. ^31^. The RNAs were extracted with Phenol-Chloroform-Isoamyl alcohol, treated with DNase I (Thermo Fisher Scientific, Product No.: EN0521) and purified on polyacrylamide gels. RNA concentrations were measured using Qubit® RNA HS Assay Kit (Thermo Scientific, Product No.: Q32852). The Z fragment RNA used here was custom synthesized (PAGE purified) by IDT DNA technologies and used without further purification.

#### Microfluidic devices and setup

Droplet-microfluidic experiments used HFE 7500 fluorinated oil (3M^TM^ Novec^TM^, Product No.:98-0212-2928-5) and a fluorosurfactant (RAN Biotechnologies, Product No.: 008-FluoroSurfactant). Devices were designed with AutoCAD software (Supplementary Data 3), printed at Selba SA. PDMS (polydimethylsiloxane) devices were fabricated by soft-lithography as described in ref. ^49^. For electrocoalescence, indium tin oxide coated glass slides were used (Delta Technologies, Product No.: CG-90IN-S215), with channels containing 3 M NaCl liquid electrodes^50^. After plasma bonding, channels were made hydrophobic by immediately flushing them with 2% 1H,1H,2H,2H-perfluorodecyltrichlorosilane (ABCR, Product No.: AB111155) in HFE 7500 with the help of syringe. Droplet-based microfluidics experiments were performed using an inverted microscope (Nikon Eclipse *Ti*) as described earlier^51^. Flows were controlled by either syringe pumps (Harvard Apparatus Inc.) or by air-pressure control pumps (MFCS™-EZ, Fluigent SA).

#### Droplet-microfluidic experiments

The complete experimental system consists of the steps **A**–**E** (Supplementary Fig. 1): (**A**) Initial emulsions production*:* Using all possible 16 WXY RNA fragments (_gMg_WXY_cNu_), 24 unique different WXY fragments combinations were prepared in separate tubes (Supplementary Table 1 and Methods section ‘Initial combinations of WXY fragments’). Experimentally, for each combination, 1 µM of respective WXY fragment(s) was heated at 80 °C for 3 min and cooled down to room temperature over 10 min (0.1 °C/sec) and mixed with hairpin RNA reporter at a final concentration of 0.03 µM. All 24 mixes were encapsulated individually in 5 pL droplets using flow focusing^52^ on a dropmaker device (Supplementary Data 3) using flow rates of 100–150 µL/h for the aqueous (RNA) and 150–170 µL/h for the oil (HFE 7500 with 2% surfactant) phases (droplet production frequency 5–10 kHz), each emulsion being collected for 5 min, corresponding to encapsulation of ~10 µL of RNA solution. All 24 emulsions were collected together in a 1.5 mL collection tube (with a PDMS cap) containing oil with 2% surfactant and mixed thoroughly. A 1.6 µM solution of Z RNA fragment in 1× *Azoarcus* reaction buffer (30 mM EPPS pH 7.4, 20 mM MgCl_2_), was folded in the same manner as WXY RNA, and encapsulated in 50 pL droplets using a 50 pL drop-making device (Supplementary Data 3), at flow rates of 350 µL/h for the aqueous (RNA) and 250 µL/h for the oil (HFE 7500 with 2% surfactant) phases, droplets being collected in a 0.5 mL collection tube (with a PDMS cap). All flows were driven by syringe pumps. (**B**) Electrocoalescence of initial emulsions: The 5 pL droplets (containing WXY fragments) and 50 pL droplets (containing Z fragment and reaction buffer) were reinjected into separate channels of a droplet fusion device (Supplementary Data 3). Both 5 pL and 50 pL droplets were spaced using HFE 7500 oil containing 2% surfactant and brought together into the same channel, where they were electrocoalesced^34,50^ using an AC electrical field (Agilent 33522 A waveform generator, sine function, 4 kHz, 400 mV, 50 Ω) amplified 10^3^ times (TREK high-voltage amplifier). Flows were regulated such that, on average, between one and five 5 pL droplets coalesce with one 50 pL droplet with a fusion frequency of ~150 Hz (Supplementary Movie 1). This resulted in ~1/10th dilution of WXY RNA concentration in the fused droplets (the Z fragment is in stoichiometric excess). All flows were driven by a pressure control system (MFCS™-EZ, Fluigent SA), and the desired fusion frequency achieved by carefully adjusting and coupling the flows with pressure values of 380–400, 350–380, 470–520 mbar for 50 pL droplet emulsion, 5 pL droplet emulsion and spacer oil channel, respectively. Electrocoalescence and collection (in a 0.2 mL tube with a PDMS cap) was performed for ~3.5 h and the emulsions were stored on ice during the entire process. To monitor the fusion frequency during the collection, fusion events were counted from videos recorded every 20 min, showing very stable fusion frequency, closely comparable to what was obtained by sequencing hairpin RNA reporters (Supplementary Fig. 4). (**C**) Incubation and splitting of droplets*:* After collection, fused droplets were incubated at 48 °C in a thermo-block for 1 h to allow the accumulation of ribozymes. In order to dilute the MgCl_2_ during the reverse transcription (RT) step, these droplets (~60–65 pL) were reinjected (spaced with HFE 7500 oil containing 2% surfactant) and split by a T-junction device^53^ (Supplementary Data 3) into smaller droplets (~5 pL) prior to the RT step. Flow rates were maintained using pressures of 800–1000 mbar and 150–250 mbar, respectively, for the oil separator channel and droplet inlet, resulting in a droplet frequency of ~150 Hz. (**D**) Droplet barcoding and RT in droplets: To sequence the RNA in each split droplet we developed a strategy combining droplet barcoding and RT in droplets, inspired by single-cell transcriptomics methods described earlier^35^. Here barcoded hydrogel beads are individually encapsulated in droplets with all the necessary reagents for RT and fused with RNA network containing droplets. The details of each step are as follows: (a*)* Barcoded hydrogel bead synthesis: Hydrogel beads were produced by co-encapsulating 10% (w/w) polyethylene glycol diacrylate (PEG-DA) 6000 (Sigma, Product No.: 701963), 1% (w/w) PEG-DA-700 (Sigma, Product No.: 455008), 400 µM of acrydite-modified dsDNA with a 4 base sticky-end (Oligo 10, top, carrying a 5’-acrydite modification, and Oligo 11, bottom, Supplementary Data 1), 10 µM of FITC-Na (Fluorescein isothiocynate), 1% (v/v) photo-initiator (2-hydroxy-2-methylpropiophenone, Sigma, Product No.: 405655) in buffer (75 mM Trizma-HCl pH 7.4, 50 mM NaCl) using flow focusing^52^ on a dropmaker device (Supplementary Data 3). 9 pL droplets were produced using flows of 150 µL/h for aqueous solution and 500 µL/h for oil (HFE 7500 with 2% surfactant). Droplets were polymerized by UV irradiation (UV omnicure AC475-365, Lumen Dynamics, ~360 mW exposure, *λ*_nm_ = 365 nm) through a PTFE tubing and collected in 5 mL tube (tubing internal diameter = 0.3 mm, distance from lamp 3 cm, tubing length = 12 cm; approximate exposure time = 47 s). Prior to UV irradiation, the complete experiment setup was maintained in the dark to avoid any spontaneous polymerization. The collected droplets were washed once with 4 mL hexane to break the emulsion and then the beads were washed three times with 4 mL binding-wash buffer (20 mM Trizma-HCl pH 7.4, 50 mM NaCl, 0.1% of Tween 20) by centrifuging at 3000 × *g* for 2 min. Beads were then filtered using Steriflip 20 µm Nylon filters (Millipore, Product No.: SCNY00020) and stored in binding-wash buffer supplemented with 1 mM EDTA. In contrast to earlier methods^35,54^, barcodes were built on the beads using a split-and-pool ligation strategy^55^ (Supplementary Fig. 2). For this, 250 µL (~10 million) of pelleted beads were washed three times with 4 mL of binding-wash buffer by centrifuging at 3000 × *g* for 2 min, and were subjected to a first ligation step. Ligation was performed on a 2 mL reaction scale with 1X T7 DNA Ligase buffer (New England Biolabs), 4 µM dsDNA adaptor with two different 4 base sticky-ends, the first being complementary to the 4 base sticky-end on the beads, and containing a BclI restriction site and the Read 2 Illumina adaptor sequence (Oligo 12, top and Oligo 13, bottom, Supplementary Data 1), 30 U/µL of T7 DNA Ligase (New England Biolabs, Product No.: M0318L), at room temperature for 25 min with agitation at 600 rpm. Beads were then washed five times with 4 mL of binding-wash buffer and pelleted after the last wash. For the next ligation step, a master-mix (1.6 mL) was prepared to contain 1× T7 DNA Ligase buffer, 30 U/µL of T7 DNA Ligase and divided into the wells of a 96-well deep well plate (16 µL per well), prefilled with 4 µL of 20 µM 1st barcode index (Index A) (Supplementary Data 1), with a different index in each well. These indexes had two different 4 base sticky-ends, the first being complementary to the free sticky-end of oligos ligated in previous step (dsDNA, Oligo 12 and 13, Supplementary Data 1). The plate was sealed and incubated at 25 °C with 600 rpm for 25 min. Then the ligase was heat inactivated at 65 °C for 10 min and the content of all the wells was pooled after adding 200 µL of binding-wash buffer containing 1 mM EDTA. After pooling, the beads were washed seven times with 4 mL of binding-wash buffer. The complete process of split-and-pool was repeated to ligate the 2nd (Index B) and 3rd (Index C) barcode indexes (Supplementary Data 1) generating a total diversity of 8.8 × 10^5^ (96^3^) barcodes. After the final pooling step, a partially double-stranded sequence was ligated to all the beads using the same ligation protocol (Oligo 14, top and Oligo 15, bottom, Supplementary Data 1). This sequence comprises a sticky-end complementary to the free sticky-end on the 3rd index, and a single-stranded 33 base long 3′-overhang at the other end (Supplementary Fig. 2b). Out of these 33 nucleotides, the first 8 are random nucleotides used as unique molecular identifiers (UMIs)^56^ and remaining 25 nucleotides, which function as a primer for RT, are complementary to the 3′ end of the WXYZ ribozyme and hairpin RNA reporter (both contain the same primer binding region). The final barcoded hydrogel bead library was washed five times with 4 mL of binding-wash buffer supplemented with 1 mM EDTA and resuspended in the same buffer. (b) Encapsulation and fusion: The barcoded beads (50 µL) were washed five times with 500 µL of binding-wash buffer by centrifuging at 11,866 × *g* for 1 min. Washed beads were then mixed with 2.5 µL of 10 mM dNTPs, 10 µL of 5× SuperScript III reaction buffer (Invitrogen), 2.5 µL of 100 mM dithiothreitol (DTT), 0.4% of Tween 20 (10%), 100 units of SUPERase۰In^TM^ inhibitor (Thermo Fisher Scientific, Product No.: AM2694) and incubated at 37 °C for 30 min to ensure diffusion of all reactants within the beads. After this, the beads were centrifuged as above and excess of liquid over the beads was removed. Then, at 4 °C, 500 units of reverse transcriptase (SuperScript III, Thermo Fisher Scientific, Product No.: 18080044) and 25 units of BclI restriction enzyme (New England Biolabs, Product No.: R0160L) were added to the beads and mixed thoroughly. These hydrogel beads together with the other reagents were encapsulated individually in ~50 pL droplets (Supplementary Movie 2), fused with RNA droplets on the same microfluidic device (Supplementary Data 3). The injection of close-packed deformable beads resulting in >99% of droplets containing a single barcoded hydrogel bead. The 5 pL RNA containing droplets and the 50 pL bead containing droplets were fused at a 1:10 ratio to ensure that, in the majority of cases, no more than one of the former is fused with one of the latter. For this, flows were pressure controlled using, respectively, 550, 225, 650, 450 mbar for hydrogel beads, 5 pL droplets, oil to control droplet spacing (HFE 7500 with 2% surfactant) and oil for beads encapsulation (HFE 7500 with 2% surfactant) resulting in a droplet frequency (50 pL) of ~100 Hz. Electrocoalescence was performed as described above. Fused droplets were collected in 0.2 mL collector tube (with PDMS cap) containing oil with 2% surfactant. (c) cDNA synthesis and extraction: The fused droplets were then incubated at 60 °C in a thermo-block for 1 h to release the DNA barcodes using the BclI restriction enzyme and perform cDNA synthesis in droplets. The emulsion was broken post incubation by adding 2 volumes of 1*H*, 1*H*, 2*H*, 2*H*-Perfluoro-1-octanol and 100 µL water. The pooled barcoded cDNAs are then isopropanol precipitated and resuspended in 40 µL of water. (**E**) Sequencing sample preparation: In order to block extension of the nonelongated primers, 20 µL of cDNA was mixed with 1× TdT reaction buffer (New England Biolabs), 0.25 mM CoCl_2_, 0.4 mM ddCTP (Roche CustomBiotech, Product No.: 12158183103) and 0.4 U/µL of Terminal Transferase (TdT, New England Biolabs, Product No.: M0315L). The reaction was carried out in 50 µL volume and incubated for 30 min at 37 °C before heat inactivation at 70 °C for 10 min. The TdT treated cDNAs were purified using magnetic beads (AMPure XP, Beckman Coulter, Product No.: A63881) following the manufacturer’s protocol. Sequencing adaptors were then appended using two sequential PCR steps (Supplementary Fig. 3a). The first PCR was performed in multiples of 20 µL volume where 2 µL of TdT treated reverse transcription reaction was mixed with 0.5 µM of Oligo 16 (also containing a 6 nucleotide stretch used as sample barcode to multiplex different samples for the sequencing run) and 0.5 µM of Oligo 17 (Supplementary Data 1) as forward and reverse primers in 1× PCR buffer (Thermo Scientific), 0.2 mM dNTPs, 0.01 U/µL of polymerase (Thermo Scientific Phusion Hot Start II, Product No.: F459) using the following protocol: initial denaturation 98 °C/30 sec, then 18 cycles of denaturing 98 °C/10 sec, annealing and extension 72 °C/1 min, and a final extension of 72 °C/5 min. PCR products were purified using AMPure XP magnetic beads and eluted in 34 µL of water. The second PCR was performed as above, but in 40 µL volume with 4 µL as PCR I purified product as template, using Oligo 18 and Oligo 19 (Supplementary Data 1) as forward and reverse primers, and using the following protocol: initial denaturation 98 °C/30 sec, then 10 cycles of denaturing 98 °C/10 sec, annealing 56 °C/30 sec, extension 72 °C/30 sec, and a final extension of 72 °C/5 min. The final sample was purified using AMPure XP magnetic beads, resuspended in 34 µL of water, analysed on TapeStation (Agilent 2200 TapeStation, using high sensitivity D1000 ScreenTape®, Product No.: 5067-5584) and quantified by Qubit® dsDNA HS Assay Kit (Thermo Scientific, Product No.: Q32854). Libraries were sequenced using the Illumina NextSeq 550 system in 2 × 150 High Output mode at the Genotyping and Sequencing Core Facility, ICM Paris (iGenSeq, Institut du cerveau et de la moelle épinière).

#### Initial combinations of WXY fragments

The 24 initial combinations of WXY fragments were computationally determined by random drawing among the 16 species. The total number of WXY fragments to distribute overall tubes (with possible redundancies) was set to 50, which was found to computationally maximize the number of distinct networks formed after random droplet fusions (10,000 iterations). Once this was fixed, network diversity was further maximized based on selecting the fragment assignment (among 100,000 random realizations) to maximize the spread (Shannon entropy) of the expected growth rate (first eigenvalue of the matrix of kinetic rates) and transient time (difference between first and second eigenvalue of the matrix of kinetic rates) distributions across networks resulting from the simulation of 10,000 random droplet fusions.

#### Sequencing data processing

Custom software was written to process sequencing data and further data analysis steps and is available at 10.5281/zenodo.4016905. The libraries were sequenced in paired-end mode with read 1 and read 2 of 180 bp and 120 bp, respectively. Read 1 was used to determine the sample barcode (used for multiplexing samples for sequencing) and the RNA molecule identity (either an *Azoarcus* ribozyme with specific IGS-tag pair or a hairpin RNA reporter). Read 2 was used to determine the UMIs^56^ and the droplet barcode. The structure of read 1 and 2 is summarized in Supplementary Fig. 3b. UMI normalization: Four meta fields were associated for each pair of reads: sample barcode, droplet barcode, UMI and RNA molecule identity (see below ‘Droplet barcode, UMI, IGS, tag, and hairpin RNA reporter identification from sequencing data’). Reads missing any of these were discarded (~30%). The reads with the same meta information were merged and the read count was added as an extra meta field. This allows to filter out noise due to sequencing errors, given that read counts resulting from such errors are significantly lower (threshold indicated on Supplementary Fig. 3e). The filtering thresholds for the minimum number of reads per UMI were determined separately for the *Azoarcus* ribozymes and hairpin RNA reporters based on the shape of the distribution of number of reads per UMI (Supplementary Fig. 3e–f). The resulting dataset is available at 10.6084/m9.figshare.c.5103962. Final data processing: Filtered UMI-normalized reads were then clustered by droplet barcode to count the number of UMIs per type of RNA molecule per droplet barcode (Supplementary Fig. 3f). For each droplet barcode, only hairpin RNA reporters where UMIs made up ≥7.5% of the total of hairpin UMIs were retained. This threshold value was set to optimally match the fusion distribution measured by video (Supplementary Fig. 3g). Within each droplet, ribozyme sequences that did not corresponding to a correct hairpin reporter were discarded. Then, only the droplet barcodes with more than 10 UMIs associated with hairpin RNA reporters and 20 with ribozymes were retained and the fraction of each species in the network was computed. As before, these thresholds were chosen to closely match the observed distribution of drop fusion determined from videos acquired during the experiments (Supplementary Fig. 3, h and i). Note that the threshold of 20 ribozyme UMIs per droplet ensures a probability of more than 89% that a species with no detected UMIs is in the 0-0.1 bin of Fig. 1d (Supplementary Fig. 3j). Imposing this probability to be >95% requires of threshold of 28 ribozyme UMIs per droplet. Measurements, which do not fulfill the latter criterion are indicated in Supplementary Data 2. Processed network compositions and mean species fractions (species fraction is the proportion of a species [WXYZ catalyst], relative to other species [WXYZ catalysts] in the network) are publicly available for all unique networks (Supplementary Data 2). To determine network yield per droplet, first we computed a droplet-specific UMI to concentration conversion rate by considering total hairpin reporter UMIs as well as number of different hairpin reporters identified. This was then used to convert total number of ribozyme UMIs into a yield value.

#### Controls for droplet-level sequencing biases

Biases in droplet-level sequencing can happen due to electrocoalescence of more than one RNA droplet to barcoding droplet and cross-overs between two different droplet barcodes during PCR steps. To quantify this, four 5 pL emulsions (A, B, C, and D) were prepared, each with: a distinct pair of hairpin RNA reporters at a final concentration of 5 nM, a set of 4 WXYZ ribozymes with the same IGS (gAg, gCg, gUg, and gGg) but in different proportions (total concentrations summing to 100 nM) and 1.6 µM of Z fragment. The concentrations of WXYZ in the four sets are as follows; Set A: 10 nM WXYZ (GA), 20 nM WXYZ (CA), 30 nM WXYZ (AA), 40 nM WXYZ (UA). Set B: 1 nM WXYZ (CC), 2.5 nM WXYZ (AC), 5 nM WXYZ (UC), 91.5 nM WXYZ (GC). Set C: 0.1 nM WXYZ (CU), 0.25 nM WXYZ (UU), 0.5 nM WXYZ (AU), 99.15 nM WXYZ (GU). Set D: 0.01 nM WXYZ (UG), 0.05 nM WXYZ (CG), 65 nM WXYZ (AG), 34.94 nM WXYZ (GG). The four emulsions were mixed and then singly fused to droplets containing hydrogel beads as described above to perform droplet-level sequencing. The data was processed as above using the same thresholding strategy. We quantified the bias as the proportion of droplets containing pure (A, B, C, or D) and compared to mixed populations (AB, AC, AD, BC, BD, CD, ABC, ABD, BCD, and ABCD, Supplementary Fig. 5d).

#### Experimental measurement of catalysis by noncovalent and covalent ribozymes

As earlier measurements^32^ of catalytic strengths between IGS/tag pairs were performed at 100 mM MgCl_2_ and different substrate concentrations than used in this study, self-assemblies were remeasured in 20 mM MgCl_2_ (present in 1× reaction buffer used here) to extract autocatalytic rate parameters (see below), using the strategy developed by von Kiedrowski^6^, as described in ref. ^32^. The concentrations of RNA fragments were kept identical to the droplet experiments (0.1 µM of WXY and 1.6 µM of Z fragment). For this, initial rates of formation of covalent WXYZ from WXY and Z fragments were measured as a function of the different concentrations of ‘doped’ covalent WXYZ ribozymes (with same IGS and tag as the WXY fragment) added at the beginning of the reaction. To facilitate the measurements, minor amounts (0.001 µM) of radiolabeled WXY fragment (γ-^32^P) were also added. These radiolabeled WXY fragments were prepared using T4 Polynucleotide Kinase (New England Biolabs) and γ-^32^P ATP (Perkin–Elmer). For each initial condition (concentration of doped WXYZ catalyst), time courses were measured to derive the initial rate of formation of covalent WXYZ from WXY and Z fragments. In accordance with previous measurements^32^, the initial rate of formation of WXYZ (*v*_0_) was found to depend linearly on the concentration of the doped WXYZ. This affine relationship can be described with the following linear equation $v_0 = \alpha x + \beta$ (Supplementary Fig. 6, b and c) where, *x* is the concentration of the covalent ribozyme, α is the slope for an IGS/tag pair, which quantifies the WXYZ synthesis by covalent ribozymes, and β is the y-intercept, which quantifies the WXYZ synthesis by noncovalent ribozymes (trans-catalysis by noncovalent complex of WXY and Z fragments)^30^. Wobble pairs (GU and UG) and one of the mismatched IGS/tag pairs (AG) were also measured and found to have negligible values for α and β (Supplementary Fig. 6c).

#### Kinetic model

In a network, covalent catalysts from species *j*, whose concentration is *x*_*j*_, contributes to the rate of formation of covalent catalysts from species *i* with the term *α*_*ij*_*x*_*j*_ while noncovalent catalysts contribute with the constant term *β*_*ij*_. *α*_*ij*_, and *β*_*ij*_ terms were measured experimentally and independently from the network experiments, for single IGS-tag pairs (Supplementary Fig. 6, b, and c). The production rate of each species is $\dot x_i = \mathop {\sum}\limits_j {\alpha _{ij} \cdot x_j + \beta _{ij}}$, resulting in a system of linear ordinary differential equations for each network depending on the IGS-tag combinations provided from the RNA fragments. This model makes two approximations. First, it assumes that noncovalent complexes are formed rapidly compared to covalent WXYZ and that variations in their concentration does not strongly affect kinetics. Vaidya et al.^30^ determined a forward rate of 0.65 μM^−1^ min^−1^ for complex formation. Given a Z fragment concentration of 1.6 μM, this gives a characteristic time of ~1 min for complex formation compared to 1 h for covalent product formation. Second, the kinetics is valid at the beginning of the reaction but does not take into account variations in substrate concentrations. In particular, Z is a shared resource between all species which may cause nonlinearities in the kinetics. However, Z is provided in excess for all network and is not strongly depleted as the median yield of reactions is 17%, to the exception the 16 member one, where is it provided in equimolar concentration with the total WXY fragments. In this case, it should be noted that Z fragment binds to all WXY fragments with the same equilibrium constant as IGS-tag variations only affect the binding between WXY and WXYZ. Consequently, Z titration is expected to affect the overall rate of total product accumulation but not to the relative production rates between species.

### Reporting summary

Further information on research design is available in the Nature Research Reporting Summary linked to this article.

## Supplementary information

Supplementary Information

Description of Additional Supplementary Files

Supplementary Data 1

Supplementary Data 2

Supplementary Data 3

Supplementary Movie 1

Supplementary Movie 2

Reporting Summary

## Data Availability

The dataset of species fractions and number of UMIs for all networks is provided as Supplementary Data 2. Raw datasets are available for download at 10.6084/m9.figshare.c.5103962.

## Code availability

Analysis code is available at 10.5281/zenodo.4016905.
