# Supplementary material for: Darwinian properties and their trade-offs in autocatalytic RNA reaction networks
Source: Nat Commun. 2021 Feb 8;12:842. doi: 10.1038/s41467-021-21000-1 (PMC7870898; doi:10.1038/s41467-021-21000-1)
Supplement: Supplementary file 1 — Supplementary Information [file 41467_2021_21000_MOESM1_ESM.pdf]

Supplementary Information for

Darwinian properties and their trade-offs in autocatalytic RNA networks

Sandeep Ameta[1,3,†], Simon Arsène[1,†], Sophie Foulon[1], Baptiste Saudemont[1], Bryce E. Clifton[2], Andrew D. Griffiths[1,*], Philippe Nghe[1,*]

[1]Laboratoire de Biochimie, ESPCI Paris, Université PSL, CNRS UMR8231, 10 Rue Vauquelin, 75005, Paris, France.

[2]School of Chemistry and Biochemistry, Georgia Institute of Technology, Atlanta, Georgia 30332-0400.

[3]Current address: Simons Centre for the Study of Living Machines, National Centre for Biological Sciences (TIFR), 560065-Bangalore, India.

*****Correspondence to: andrew.griffiths@espci.fr, philippe.nghe@espci.fr.

[†]Sandeep Ameta and Simon Arsène contributed equally to the work.

This PDF file includes:

Supplementary Methods

Supplementary Notes

Supplementary Figs. 1 to 13

Supplementary Tables 1 to 2

Supplementary References 1 to 3

Supplementary Methods

Quality control of hydrogel beads. The quality of the barcoded beads was assessed by analysing the percentage of full-length barcodes in the final library as well as the diversity of barcodes on beads. The percentage of full-length barcodes was analysed by subjecting a small portion of the beads to restriction endonuclease treatment (BclI, New England Biolabs, Product No.: R0160L). The barcodes have unique BclI site in the first common adaptor which is also used to release the barcodes from beads during reverse transcription in droplets (as described in the Methods section). The beads were centrifuged and supernatant was analyzed on TapeStation (Agilent 2200 TapeStation, using high sensitivity D1000 ScreenTape®, Product No.: 5067-5584). On average ~70% of the total oligonucleotide released contained full-length barcodes (Supplementary Fig. 2c).

To check the diversity of barcoded primers on beads, single beads were sorted into wells, containing 4 µL of water, of a 96-well plate using a fluorescence activated cell sorter (FACS; ARIA III, BD Biosciences, San Diego, at the Plate-forme de Cytometrie et d'Immuno-biologie, CYBIO, Institute Cochin, Paris). Then a reverse transcription reaction, in a total volume of 10 µL, was performed inside the plate using the same protocol and conditions as described above, but using a row-specific hairpin RNA reporter (~ 0.1 µM) as template. This generate cDNAs with the bead barcode coupled to the row-specific hairpin reporter sequence. After 1 h incubation, 2 µl of the RT reaction from each well was transferred to a new plate and two sequential PCRs were performed to prepare samples for sequencing. The first PCR was performed in a volume of 20 µL using a column-specific forward primer (Oligo 16, sample barcodes 1 to 12, Supplementary Data 1) and a common reverse primer (Oligo 17, Supplementary Data 1). PCR products were purified separately from each well using 1.2 equivalent of AMPure XP magnetic beads (Beckman Coulter, Product No.: A63881) and re-suspended in 20 µL of water. 4 µL of purified products were used

for second 40 µL PCR, using common forward and reverse primers (Oligo 18 and 19 respectively, Supplementary Data 1) for all the wells. For both PCRs, the thermocycling was done as described for the droplet experiments. After the second PCR, products were analysed on 1% agarose gels. 71/96 wells showed amplification. Then, 1 µL of PCR products from the positive wells were pooled, purified using 1.2 equivalent of AMPure XP magnetic beads (Beckman Coulter, Product No.: A63881), and sequenced on a HiSeq 4000 system (2*150 High Output mode at BGI Sequencing, Hong-Kong, China). The sequencing data was processed as described above. No filter based on the number of reads per UMIs was applied. UMI-normalized reads were associated to a bead using row and column-specific barcoding (rows coded by specific hairpin RNA reporter for each row; columns coded barcode introduced during the first PCR). For each bead (n=71), we computed the percentage of the most common barcode and found that all expect 3 beads had a mean value of 95.3 ± 2.3% (Supplementary Fig. 2d). The three beads with values significantly lower (around 50%), contained two dominant barcodes, each comprising ~ 50% of the reads, consistent with the presence of two beads in these wells.

Duplication of IGS. In order to sequence the 'IGS' at the 5' end of the ribozyme (WXYZ) RNA (without erasing it with the forward primer during PCR and to avoid biases from a ligation-based strategy), the central nucleotide of the IGS sequence was duplicated within WXY fragments. This was achieved by mutating the 25th nucleotide of the WXY fragment ('A' in the wild-type ribozyme, as used in the study of Vaidya *et al.* [1]) to either C, U, or G with complementary base-pairing mutations at the 7th nucleotide using site-directed mutagenesis. For this, wild-type DNA template (~ 0.25 pg/µL) was amplified by PCR using forward and backward M25 mutation primers (Oligo 1 to 4 and Oligo 5, respectively, Supplementary Data 1). The respective PCR product was cloned in pJET2.1 vector using CloneJET PCR cloning kit (Thermo Scientific, Product No.: K1231)

following the manufacturer's protocol. The cloned products were transformed in *E. coli* (Top10 Chemical competent cells, Thermo Scientific, Product No.: C404010), incubated on LB agar-ampicillin plates, positive colonies were selected, and plasmids were isolated (using NucleoSpin® Plasmid kit from Macherey-Nagel, Product No.: 740588.10) and sequenced by Sanger sequencing (GATC Biotech). The clones with the correct sequence were used as template in PCR reactions to obtain dsDNA templates for the *in vitro* transcription in order to generate all 16 combinations of WXY RNA fragments ($_{gMg}WXY_{cNu}$).

Quality control of WXY RNA fragments. In order to check whether the IGS at 5'end of WXY fragments corresponds to the duplicated IGS mutation at 25th position (and corresponding base-pair mutation at 7th position), all the $_{gMg}WXY_{cNu}$ fragments used in the study were individually sequenced. For this, all 16 WXY RNA fragments were poly(A) tailed, ligated with an RNA adaptor at their 5'end, converted to cDNA, appended with sequencing adaptors, and sequenced. For poly(A) tailing, 2.5 µM of each WXY RNA (in a separate reaction) was mixed with 1X reaction buffer (50 mM Tris-HCl pH 7.9, 250 mM NaCl, 10 mM $MgCl_2$), 2 mM ATP, 50 U/µL of *E. coli* poly(A) polymerase (New England Biolabs, Product No.: M0276S) and incubated at 37°C for 40 min. After heat inactivation (70°C, 10 min) and isopropanol precipitation, the RNA were re-suspended in water, mixed with 1X reaction buffer (10 mM Tris-HCl pH 8.0, 5 mM $MgCl_2$, 100 mM KCl, 0.02% Triton X-100, 0.1 mg/mL BSA), 0.1 U/µL of phosphatase enzyme (FastAP, Thermo Fisher Scientific, Product No.: EF0652) and incubated at 37°C for 1 h to remove the 5'-triphosphate from RNA. After heat inactivation (70°C, 10 min), the dephosphorylated RNA reaction (20 µL) was subjected to phosphorylation (adding 5'-monophosphate) reaction (in 40µL volume) by adding 1X reaction buffer (50 mM Tris-HCl pH 7.6, 10 mM $MgCl_2$, 5 mM DTT, 0.1 mM spermidine), 2 mM ATP, 0.25 U/µL of T4 Polynucleotide Kinase (Thermo Fisher Scientific,

Product No.: EK0031) and incubating at 37°C for 1 h. After heat inactivation (70°C, 10min), RNAs were purified on AMPure XP magnetic beads (Beckman Coulter, Product No.: A63881). The mono-phosphorylated RNAs (10 µL) were ligated (in 20 µL volume) to an RNA adaptor (Oligo 21, Supplementary Data 1) by mixing with 1X reaction buffer (50 mM Tris-HCl pH 7.5, 10 mM $MgCl_2$, 1 mM DTT), 2 mM of ATP, 3% of PEG8000, 1 µM of adaptor RNA, 2.2 U/µL of T4 RNA ligase 1 (New England Biolabs, Product No.: M0204S) and incubating at 16°C overnight. After heat inactivation, RNAs were purified on AMPure XP magnetic beads (Beckman Coulter, Product No.: A63881) and reverse transcribed. For reverse transcription (RT), purified ligated RNAs (10 µL) were mixed with 1X reaction buffer (50 mM Tris-HCl pH 8.3, 75 mM KCl, 3 mM $MgCl_2$), 0.5 mM of each dNTP, 5 mM of DTT, 2.5 µM of RT primer (Oligo 22, Supplementary Data 1), 10 U/µL of Superscript III enzyme (Thermo Fisher Scientific, Product No.: 18080085) in reaction volume of 10µL and incubated at 55°C for 1 h. After RT, cDNAs were purified using AMPure XP magnetic beads (Beckman Coulter, Product No.: A63881) and amplified by PCR to append sequencing adaptors (as described in the Methods section, with Oligo 23, 17 as forward and reverse primer, respectively in PCR I and Oligo 18, 19 as forward and reverse primer, respectively in PCR II, Supplementary Data 1). Final amplicons were analyzed by 2x150bp pair-end sequencing (microMiSeq, Institute Curie High Throughput Sequencing Platform, Paris). For each WXY fragment, the corresponding reads were first analyzed to extract the sequence of the IGS and the two internal mutations (at positions 7 and 25) coding for the IGS. The results show that ~97% of the IGS correctly matched the expected nucleotides at positions 7 and 25.

Droplet barcode, UMI, IGS, tag and hairpin RNA reporter identification from sequencing data. _Droplet barcode identification_: The droplet barcode is composed of three 16 nucleotide long variable regions denoted as ‘indexes’ separated by 4 nucleotide long constant regions denoted as

‘linkers’ (Supplementary Fig. 2b). First the linker positions are searched using a sliding window approach around their expected positions in a window of 5 bp and allowing a maximum Hamming distance of 1. The sequence in between two identified linkers is then extracted and aligned against the 96 possible variants (Supplementary Data 1) for the corresponding index using Bowtie 2 version 2.2.9 with the following parameters: --very-sensitive --reorder -t. Alignments with a mapping quality lower than 20 were discarded. This gives the identity of the 3 indexes composing the droplet barcode for each read.

UMI identification: To identify the UMI, first the position of the constant 15 bp sequence before the UMI is searched around its expected position in a window of 20 bp, allowing a maximum Hamming distance of 2. If a position is found and, if the last 3 bp exactly match the end of the expected constant 15 bp sequence, then the following 8 bp are extracted as the UMI for the read.

IGS identification: Position 25 in the *Azoarcus* ribozyme, which corresponds to position 31 in read 1, codes for the IGS. To identify the base at position 31 of read 1, an upstream constant 7 base region (which starts at position 23) is searched in a window of 10 bp allowing a maximum Hamming distance of 2. If a position is found, and if the 3 bases after this stretch are in the following format: ‘CMA’ then ‘M’ is extracted as the IGS for the read.

tag identification: tag identification is similar to IGS identification. The constant region 10 bases upstream of the tag (‘CNU’) in read 1 is searched around its expected position in a window of 10 bp allowing a maximum Hamming distance of 2. If a position is found and if the 3 bases, located 10 bp after this position, are in the following format: ‘CNU’ then N is extracted as the tag for the read.

Hairpin RNA reporter identification: The hairpin RNA reporter sequence contains a variable 4 nucleotide region in the loop region which codes for its identity. To test whether a read corresponds

to a hairpin reporter, the constant 10 bases region before and after the variable region are searched around their expected positions in windows of 10 bases and allowing a maximum Hamming distance of 2. If the two sequences are found the read is declared to be a hairpin RNA reporter read. The variable 4 nucleotides between these two constant regions are then used to assign the hairpin reporter identity. This sequence is compared to the list of possible variable region and the closest one in terms of Hamming distance (but not more distant than 1) is taken as the hairpin RNA reporter identity.

Determination of network composition in each droplet. To determine which *Azoarcus* network is present in each droplet, the ribozyme composition (obtained with the WXYZ reads where 'IGS' and 'tag' are identified, see sections above) is compared with the expected network structure from molecular hairpin RNA reporters. For this, the 24 initial 5 pL droplet emulsions are additionally barcoded with these hairpin RNA reporters as an independent measurement of expected network composition inside droplets after fusion. This allows thresholds to be chosen in sequencing data analysis, to correct compositional data, and to assign the network composition in each droplet. These molecular RNA reporters are small hairpin RNAs which contain a 4 nucleotide long unique barcode in the loop region and share the same primer binding site as *Azoarcus* ribozyme in the stem part so that they can be specifically barcoded per droplet and sequenced together with WXYZ ribozymes.

The 24 hairpin RNA reporters were produced by *in vitro* transcription as described in the Methods section. Templates for transcription were generated by PCR using 0.0025 µM of synthetic ssDNA as template (Supplementary Data 1) and 0.5 µM of each forward and reverse primer (Oligo 20, and 5, respectively Supplementary Data 1). The absence of interference with the *Azoarcus* recombination was verified by performing reactions in the presence or absence of hairpin RNAs.

For this, 0.5 µM each of WXY (with 'gAg' as IGS and 'cUu' as tag) and Z RNA fragments were mixed with 1X reaction buffer (30 mM EPPS pH 7.4, 20 mM $MgCl_2$). This reaction was carried out with or without 36 nM of hairpin RNA reporter and incubated for 6 h. Sample were withdrawn at regular time intervals and analysed on a 12% denaturing polyacrylamide gel. The formation of WXYZ ribozymes were calculated from the band intensity using ImageJ software (https://imagej.nih.gov/ij/). The results show that there is no impact of these RNA reporters on the *Azoarcus* recombination reactions as the time-courses are indistinguishable.

Growth and composition measurement of representative examples of networks by gel-electrophoresis and bulk-sequencing. To perform an independent comparison of growth and composition of the network observed in the droplet dataset, three representative networks, one low-yield, one mid-yield, and one high-yield, of covalent WXYZ, identified from droplet sequencing data (Fig. 2a), were analyzed in bulk. Time-courses were measured for all three networks (in triplicates) and WXYZ formation was analyzed by gel-electrophoresis. For this, respective WXY RNA fragments (low-yield: CA, CC, CU, GU; mid-yield AC, CU, UG, GA; high-yield: AC, AG, GC, GG) at 0.1 µM each with 1.6 µM of Z fragment (same as in droplet experiments) were mixed, heated 80°C for 3 min, cooled down to room temperature over 10 min (0.1°C/sec), mixed with reaction buffer (30 mM EPPS, 20 mM $MgCl_2$) and incubated at 48°C for 4 h. The reaction scale was 40 µL. At different time-points (0, 15, 30, 60, 120, 180, 240 min), 4 µL reaction mix was quenched with 2X loading buffer (containing 120 mM EDTA, along with xylene cyanol and bromophenol blue as gel tracking dyes) and frozen at -20°C. All time-points were then analyzed by gel-electrophoresis using 12% denaturing polyacrylamide gels (in 1X TBE), stained with SYBR Green (Thermo Fisher Scientific, Product no.: S7563), and imaged using UV-transillumination. WXYZ formation was calculated by analyzing the product band intensities

using ImageJ (https://imagej.nih.gov/ij/index.html). To know the composition for the network at 1 h (the same incubation as in the droplet experiments) bulk sequencing was performed. For this, a separate reaction for each network (using same protocol and concentration above) was set up and at 1 h, 5 µL of reaction was take-out and subjected to reverse transcription (RT). For RT, the reaction was mixed with 0.5 mM dNTP, 1X RT Buffer (Superscript III reaction buffer, Invitrogen), 5 mM DTT, 2.5 µM of Oligo 24 (Supplementary Data 1) as RT primer, and 10 U/µL of RT enzyme (Superscript III, Invitrogen) in a 20 µL reaction, and incubated at 55°C for 1 h. After incubation, the RT products were purified using 2 equivalents of AMPure XP magnetic beads (Beckman Coulter, Product No.: A63881), re-suspended in 20 µL of water and 10 µL subjected to RNaseH treatment to prepare RNA-free cDNAs. For this, 10 µL of purified RT reaction was mixed with 1X RNase H buffer (NEB) and 0.5 U/µL of RNase H enzyme (NEB) in a 20 µL reaction and incubated at 37°C for 20 min followed by heat inactivation at 65°C for 20 min. cDNAs were purified using 2 equivalents of AMPure XP magnetic beads (Beckman Coulter, Product No.: A63881) and re-suspended in 20 µL of water. RNA-free cDNAs were then diluted to 10^5 times and 4 µL of this dilution from each network sample was subjected to two-step PCR process using the same primers and protocols used for the droplet experiments as described in Methods section (in the ‘Droplet microfluidic experiments’ sub-section). Sequencing samples were also prepared in the same way as for the droplet experiments and multiplexed with other sequencing samples for sequencing on an Illumina NextSeq 500 system in 2*150 Mid Output mode at the Genotyping and Sequencing Core Facility, ICM Paris (iGenSeq, Institut du Cerveau et de la Moelle Épinière). The sequencing data was processed and analyzed similarly as described above (see the Methods section ‘Sequencing data processing’) to extract species fractions.

Supplementary Note

Analytical expressions for species-level perturbation. We consider the addition of a species a to the network G resulting in network $G' = (V', E')$ as $V' = V + \{a\}$. Fraction of species v in G', $y_v^{G'}$ can be taken relative only to the species in V (i.e. excluding a giving $y_v^{G'}|_V = \frac{y_v^{G'}}{\sum_{u \in V} y_u^{G'}}$. Species v response to the perturbation induced by the addition of species a to network G can be measured by $p_{G \to G'}^{v} = y_v^{G'}|_V - y_v^{G}$. Under the assumption that $y_v^G \sim \frac{D_v^{in,G}}{\sigma_G}$, one obtains:

$$p_{G \to G'}^{v} = \frac{\frac{D_v^{in,G'}}{\sigma_{G'}}}{\sum_{u \in V} \frac{D_u^{in,G'}}{\sigma_{G'}}} - \frac{D_v^{in,G}}{\sigma_G}$$

$$p_{G \to G'}^{v} = \frac{D_v^{in,G} + e_{a \to v}}{\sigma_G + D_a^{out,G}} - \frac{D_v^{in,G}}{\sigma_G}$$

$$p_{G \to G'}^{v} = \frac{e_{a \to v} - \frac{D_v^{in,G} D_a^{out,G}}{\sigma_G}}{\sigma_G + D_a^{out,G}}$$

Here, $e_{a \to v}$ is the weight of the directed edge between the new species a and species v. We can now distinguish two cases depending on whether or not the species v is a target for the new species a. If v is a target for a, then $e_{a \to v} = e$, and we can introduce the normalized in-degree $m_v = \frac{D_v^{in,G}}{e}$, the normalized sum of all the weights in the network $\sigma_e = \frac{\sigma_G}{e}$ and $n = \frac{D_a^{out,G}}{e}$, which is the number of targets of a in the network. Then dividing the obtained $p_{G \to G'}^{v}$ expression by e gives:

$$p_{G \to G'}^{v} = \frac{1 - n \, \frac{m_v}{\sigma_e}}{\sigma_e + n} \ (i)$$

Because $\sigma_G \geq n m_v e$, in this case $p_{G \to G'}^{v} \geq 0$.

If v is not a target for a, then $e_{a\to v} = 0$ and in this case $m_v = \frac{D_v^{in,G}}{e_v}$ where e_v is the weight of an incoming edge to v. We obtain in this case:

$$p_{G\to G'}^{v} = \frac{-n\,\frac{m_v}{\sigma_e}}{\sigma_e +\ n} \cdot \frac{e_v}{e}\ (ii)$$

In this case, it is clear that $p_{G\to G'}^{v} \leq 0$.

Analytical expression for network-level perturbation. Let $V_a = \{v \in V, e_{a\to v} > 0\}$ be the set of species in G for which a is a catalyst and $V_a^* = \{v \in V, e_{a\to v} = 0\}$ the set of species for which it is not. We assume that a is a catalyst for at least one species ($n > 0$) and that there is at least one link in G ($\sigma_G > 0$). The perturbation of the network composition is:

$$p_{G\to G'} = \sum_{v\,\in V} |p_v^{G\to G'}|$$

Using expressions (i) and (ii) derived above, we obtain:

$$p_{G\to G'} = \sum_{v\,\in V_a} (\frac{1-n\,\frac{m_v}{\sigma_e}}{\sigma_e +\ n}) + \sum_{v\,\in V_a^*} \frac{n\,\frac{m_v}{\sigma_e}}{\sigma_e +\ n} \cdot \frac{e_v}{e}$$

$$p_{G\to G'} = \frac{n}{\sigma_e +\ n}\left(1 + \frac{1}{\sigma_e}(-\sum_{v\,\in V_a} m_v + \frac{1}{e}\sum_{v\,\in V_a^*} m_v e_v)\right)$$

Let m be the number of species in G with the same IGS as a, in other words, these m species and a are similar catalysts. It follows that $m = \sum_{v\,\in V_a} m_v/n$ and that $\frac{1}{e}\sum_{v\,\in V_a^*} m_v e_v = \sigma_e - nm$. This results in the expression presented in the main text:

$$p_{G\to G'} = 2n\frac{1-n\,\frac{m}{\sigma_e}}{\sigma_e + n}\ (1)$$

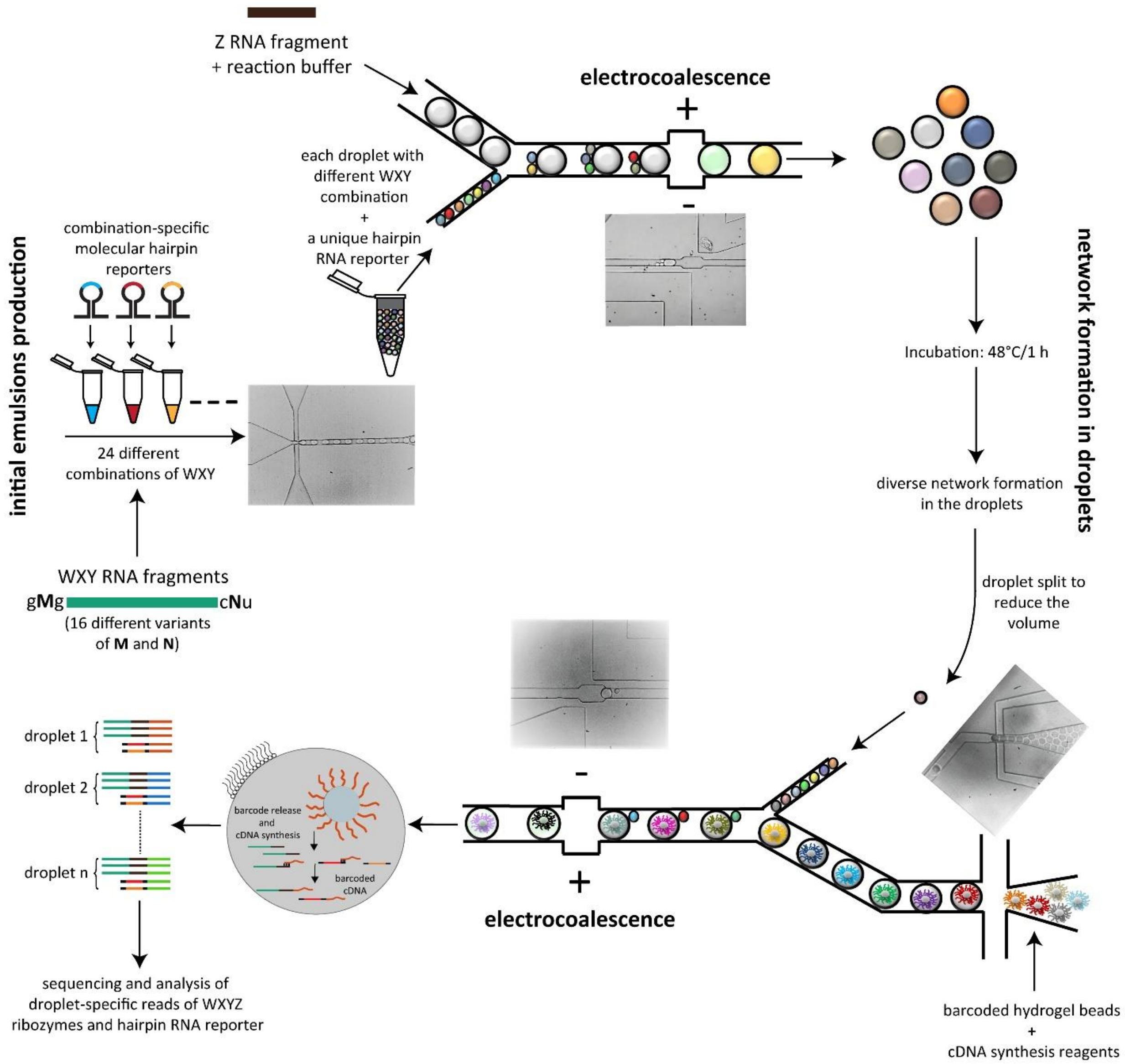

Supplemenatry Figure 1. Schematic of the complete experimental set-up. The different steps involved in the experiment. See Methods for details. On a first microfluidic device, 24 different initial emulsions, comprising 5 pL droplets, were prepared, each containing a unique combination of WXY fragments (Supplementary Table 1), and a unique molecular hairpin RNA reporter. These emulsions were collected in a single tube (1.5 mL) and mixed thoroughly. These 5 pL droplets were then re-injected into a second microfluidic device, where they were fused by electrocoalescence with 50 pL droplets, produced on a separate chip, containing Z fragment and

the reaction buffer. The pairing of 5 pL and 50 pL droplets was controlled such that one to five 5 pL droplets fuse with one 50 pL droplet. After fusion, the resulting emulsion, comprising 65 pL droplets, was incubated at 48°C for 1 h, and then split into 5 pL droplets on a third microfluidic device to reduce the volume. These droplets were re-injected into a fourth microfluidic device, where they were fused with 50 pL droplets, produced on the same chip, containing single hydrogel beads carrying barcoded cDNA primers, and reverse transcription reaction reagents. The ratio of 5 pL to 50 pL droplets was kept low (1:10) such that only one 5 pL RNA droplet was fused with one 50 pL droplet. The resulting emulsion was incubated at 60°C for 1 h to allow release of barcoded primers from the beads and cDNA synthesis. Then, the emulsion was broken, the barcoded cDNA was amplified by PCR to append sequencing adaptors, and sequenced.

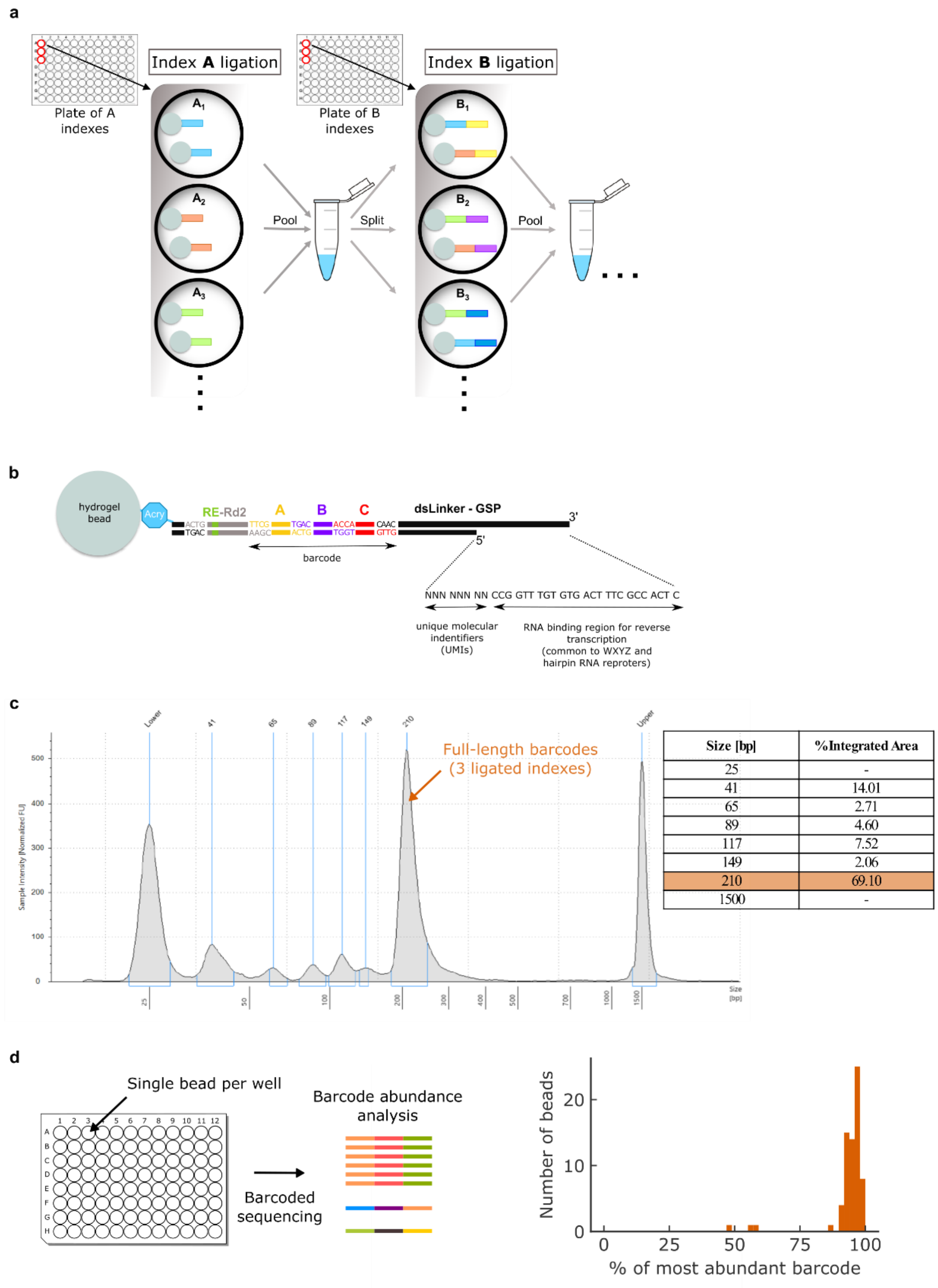

Size [bp]	%Integrated Area
25	-
41	14.01
65	2.71
89	4.60
117	7.52
149	2.06
210	69.10
1500	-

Supplementary Figure 2. Barcoded hydrogel beads. a. Synthesis of barcoded hydrogel beads using a split and pool ligation strategy (details in Methods). On each bead, the clonal population

of barcodes is built by successive ligation of indexes in one of the wells of 3 successive 96-well plates, in which each well contains a different index. Between each ligation step, beads are pooled before being distributed randomly in the 96 wells of the next plate. After the ligation of three indexes (A, B and C), a common oligo containing a linker (double stranded), UMIs (8 nt, single stranded), and the RNA binding region for reverse transcription (25 nt, single stranded) is ligated. **b.** Final structure of a barcode: linker, restriction site (RE), Read 2 Illumina sequence (Rd2), 3 barcode indexes (A, B and C) separated by linker regions, UMI and cDNA primer. The sequences of the ligated 4 base sticky-ends in the linker regions are indicated. **c.** Capillary electrophoresis (TapeStation, Agilent) data of the barcoded primers released from beads by restriction digestion. The length at the main peak (~70%) corresponds to fully ligated barcodes with three indexes. Note that the actual length of full barcodes is 152 bp compared to the length indicated by TapeStation (210 bp) due to barcodes being partially single-stranded. See Methods for the details. **d.** Distribution of percentage of the most abundant barcode sequence per bead is plotted for n=71 beads obtained by sequencing of barcodes released from single beads in microplate wells. See Supplementary Methods section for the details.

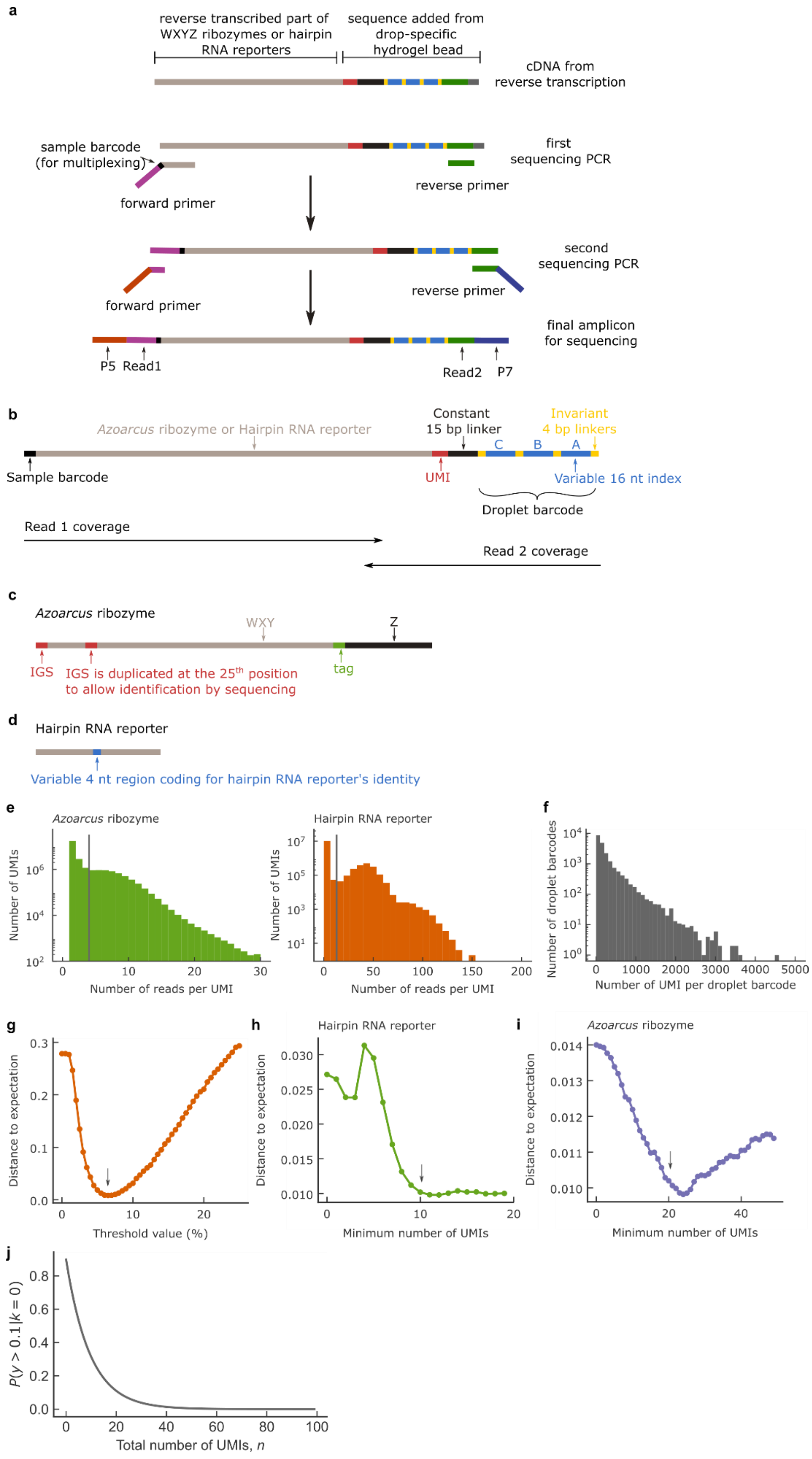
a
reverse transcribed part of WXYZ ribozymes or hairpin RNA reporters
sequence added from drop-specific hydrogel bead
cDNA from reverse transcription
sample barcode (for multiplexing)
first sequencing PCR
forward primer
reverse primer
second sequencing PCR
forward primer
reverse primer
final amplicon for sequencing
P5
Read1
Read2
P7
b
Azoarcus ribozyme or Hairpin RNA reporter
Constant 15 bp linker
Invariant 4 bp linkers
C
B
A
Sample barcode
UMI
Variable 16 nt index
Droplet barcode
Read 1 coverage
Read 2 coverage
c
Azoarcus ribozyme
WXY
Z
IGS
IGS is duplicated at the 25th position to allow identification by sequencing
tag
d
Hairpin RNA reporter
Variable 4 nt region coding for hairpin RNA reporter's identity
e
Azoarcus ribozyme
Number of UMIs
Number of reads per UMI
Hairpin RNA reporter
Number of UMIs
Number of reads per UMI
f
Number of droplet barcodes
Number of UMI per droplet barcode
g
Distance to expectation
Threshold value (%)
h
Hairpin RNA reporter
Distance to expectation
Minimum number of UMIs
i
Azoarcus ribozyme
Distance to expectation
Minimum number of UMIs
j
P(y > 0.1|k = 0)
Total number of UMIs, n

Supplementary Figure 3. Sequencing and analysis. a. Schematic of PCR steps to append sequencing adaptors (see Methods). In the first step, barcoded cDNAs are amplified with a gene-specific forward primer, which also contains a barcode for multiplexing different samples for sequencing, and a common read 2 reverse primer. The PCR products from first PCR are then subjected to a second PCR to add adaptors compatible with Illumina sequencing platforms. **b-c** Sequencing read structures **b.** Generic paired-end read structure. **c.** Detailed structure of the reads for *Azoarcus* ribozymes and for hairpin RNA reporters. **d.** Structure of molecular hairpin RNA reporters, which share the same 5' and 3' regions as WXYZ to allow amplification. These hairpins have a variable loop region of 4 nucleotide serving as barcode. **e.** Distribution of number of UMIs as a function of the number of reads per UMI for reads associated with *Azoarcus* ribozymes or hairpin RNA reporters. The threshold, indicated by the gray line, is determined by the intersection of the "noise" distribution with very low number of reads per UMI and the "signal" distribution with higher mean number of reads per UMI. UMIs with less reads than this threshold were discarded. Note that x-axis scale for the plots is different because of the more efficient amplification of hairpin reporters during PCR due to their smaller size. **f.** Distribution of number of UMIs (ribozyme and hairpin) per droplet barcode. **g-i** Thresholds on the minimum number of UMIs per droplet barcode have been determined to optimize the correspondence between the statistics of the number of droplets fused as measured by video microscopy and by hairpin sequencing. This correspondence is measured as a 'distance to expectation', which is the Euclidian distance between the distribution of the number of hairpin RNA reporters per droplet barcode once the three thresholds have been applied, and the distribution of droplets at the fusion step measured by video acquisition. The three thresholds chosen for subsequent data analysis are shown as grey arrows: **g.** the minimum percentage of total hairpin RNA reporter UMIs used to declare the

corresponding hairpin reporter to be part of the coding set for a given droplet barcode (chosen value = 7.5); **h.** the minimum number of hairpin RNA reporter UMIs for a droplet barcode to be included in the final dataset (chosen value = 10), and; **i.** the minimum number *Azoarcus* ribozyme UMIs for a droplet barcode to be included in the final dataset (chosen value = 20). **j.** Probability that a species fraction $\boldsymbol{y}$ is >0.1 (i.e. is not in the first bin in Fig. 1d) when no UMIs were detected for that species ($\boldsymbol{k = 0}$) in a droplet. $\boldsymbol{P(y > 0.1 | k = 0) = \int_{0.1}^{1} (1 - y)^{n} dy \Big/ \int_{0}^{1} (1 - y)^{n} dy}$ assuming independent and identically distributed trials and a uniform prior for $\boldsymbol{y}$, $\boldsymbol{n}$ being the total number of *Azoarcus* ribozyme UMIs for the droplet barcode.

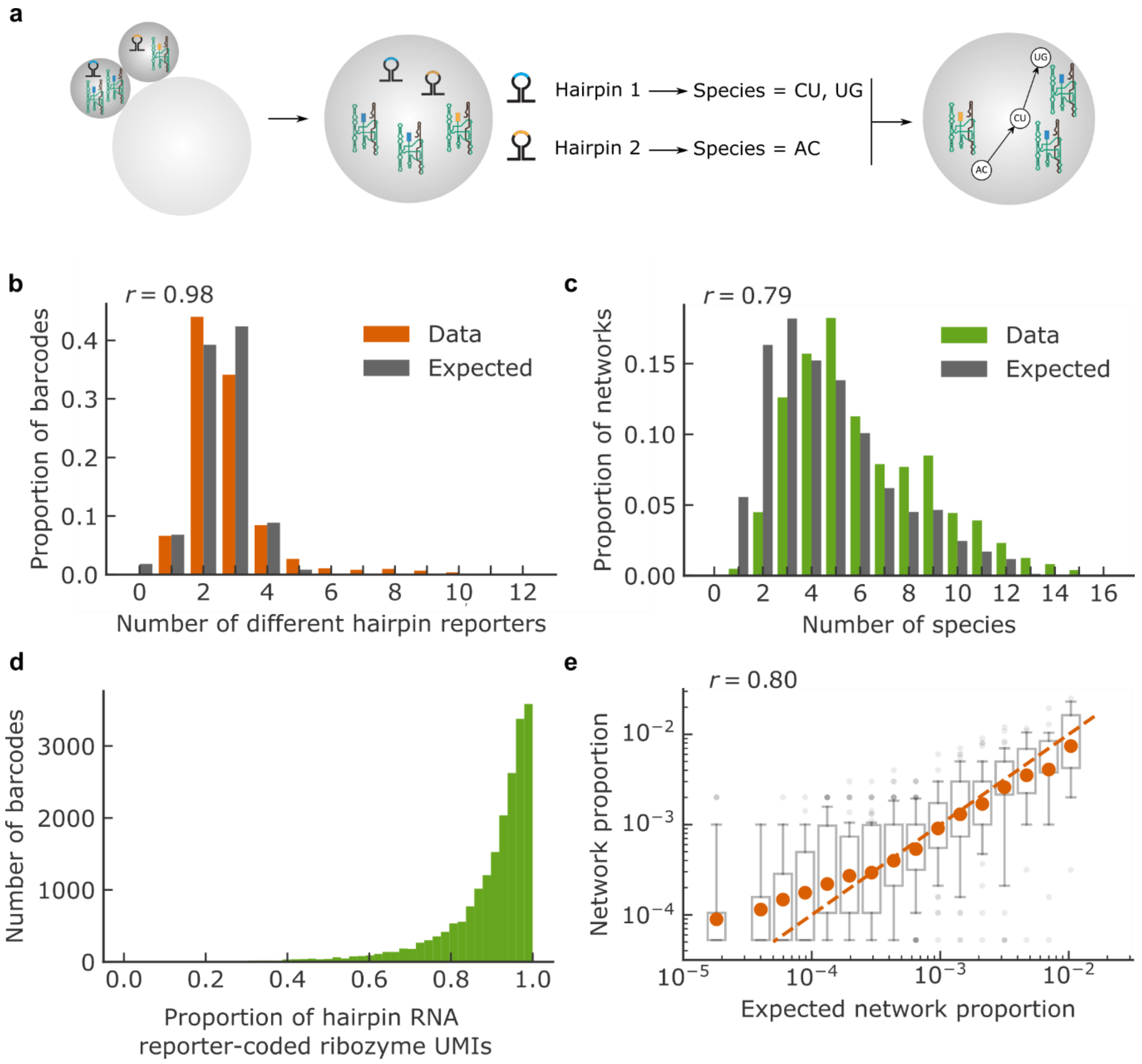

Supplementary Figure 4. Identification of the network structure at the droplet level using hairpin RNA reporters. a. Schematic illustrating identification of a network structure using hairpin RNA reporters. The hairpin RNAs where the UMI counts make up ≥7.5% of the total number of UMI counts for hairpin RNAs are considered as part of the coding set. This can be used to derive network structure in addition to species identification using an internal mutation in WXYZ ribozyme (Supplementary Fig. 3c and Methods). The number of UMIs for each species of the identified network structure gives the species fraction. **b.** In orange, measured distribution of the number of hairpin RNA reporters per droplet barcode in the final dataset. In dark grey,

distribution of number of 5 pL droplets (containing initial WXY emulsions) fused with a 50 pL droplet (containing reaction buffer and Z) measured by video acquisition (Methods). Pearson's correlation coefficient is reported (p=3.2e-9, n=13). **c.** In green, distribution of number of species per network per droplet barcode in the final dataset. In dark grey, expected distribution from simulating the library fusion step with the same number of droplets as in the data and with the fusion frequencies measured by video acquisition (Methods). Pearson's correlation coefficient is reported (p=2.0e-4, n=17). **d.** Histogram of distribution of the proportion of hairpin RNA reporter-coded ribozyme UMIs, defined as the ratio between the total numbers of UMIs associated with species identified by the hairpin reporters and the total number of UMIs for *Azoarcus* ribozyme identified by the internal mutation (Methods) for the same droplet barcode. A value of 1 is reached when all ribozyme UMIs predicted by the set of hairpin reporters are identified. **e.** Correlation between the measured and expected network proportions. Network proportion is calculated as the ratio between the number of replicates of a given network and the total number of replicates for all networks. Expected network proportion is obtained by simulating the library fusion step with 50,000 droplets mimicking the experimental set-up using the fusion frequencies measured by video acquisition. About 1/3 of the networks in the data do not have any replicate in the simulation and are excluded from the analysis. Bins with fewer than 10 points were discarded. The box extends from the lower to upper quartile values of the data, with a dot at the mean. The whiskers extend from the 5th percentile to the 95th. Flier points are those past the end of the whiskers. The dotted orange line is the identity line. Pearson's correlation coefficient is reported (p<1e-5, n=2160).

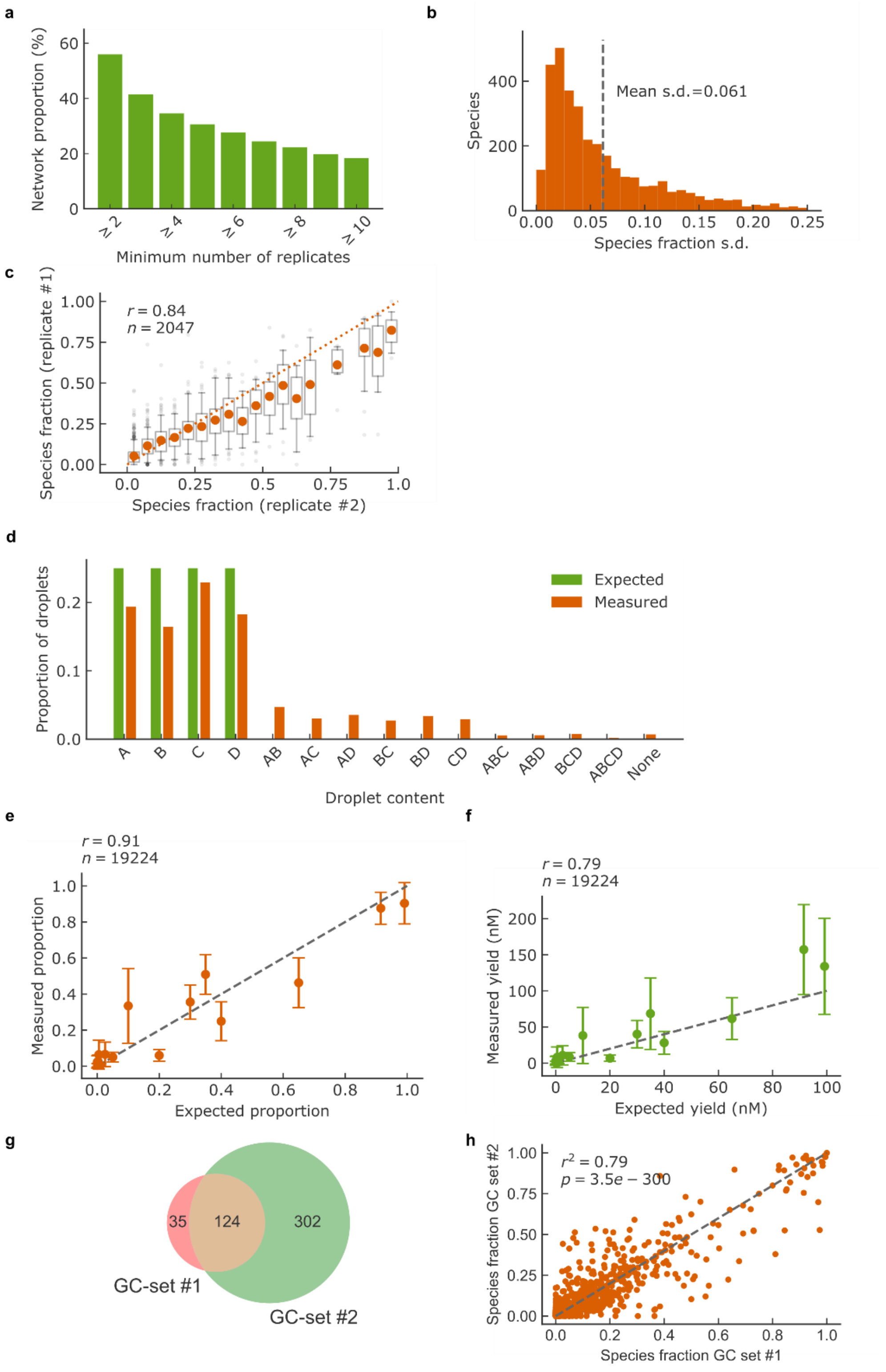

Supplementary Figure 5. Measurement precision. a. Proportion of unique networks having replicates. 56.0% of the networks in the final dataset have at least duplicates and 18.3% of them

have at least 10 replicates. **b.** Distribution of species fraction standard deviation where standard deviation is measured from the networks with at least 5 replicates. The dotted grey line is the mean of the distribution of standard deviation. The result indicates a mean precision of ~6% in species fractions. **c.** The complete experiment (Methods, Supplementary Fig. 1) was replicated and species fractions in both replicates were measured. Species fractions measured in one experimental replicate are plotted against the other (n=2047 species). Data is restricted to the common set of networks present in the two replicates. Data points are binned in 20 linearly spaced bins according to their x-axis component. Bins with fewer than 10 points were discarded. The box extends from the lower to upper quartile values of the data, with a dot at the mean. The whiskers extend from the 5th percentile to the 95th. Flier points are those past the end of the whiskers. The dotted orange line is the identity line. Pearson's correlation coefficient is reported ($p<1e-5$, n=2047). These technical replicates demonstrated high repeatability (r=0.84 between species fractions). **d-f** Control for cross-talk and quantification bias during droplet barcoding and sequencing. Barcoded sequencing was performed on a mix of four emulsions (A, B, C and D) each containing a mixture of ribozymes in known proportions as well as a pair of specific hairpin RNA reporters for identification (Methods). **d.** Measured proportions of each population of droplets (orange) and expectations (green). The result indicates 87% of sequenced droplet contained a single set of ribozymes which demonstrate a low cross-talk between droplets. **e-f.** Measured against expected ribozyme concentrations plotted as **e**, proportion and **f,** absolute values (in nM). Aggregating data from the four emulsions show high correlation (r = 0.91 and r = 0.79) and rule out biases of the droplet level sequencing. Each dot corresponds to a ribozyme in one of the emulsions (4 ribozymes in each of the 4 emulsions). Data are represented as mean values ±1 s.d.. Grey dotted line is the identity line. Pearson correlation coefficient $p<1e-5$ in all cases. **g-h.** GC redundancy as an

additional internal control: the initial fragment pools contain two identical sets of GC WXY fragments, each labeled with a different hairpin barcode (Supplementary Table 1, combination # 5 and 17). **g.** Venn diagram showing the fragment composition overlap between the two sets of networks comprising one of the GC pool, as obtained after droplet fusion. **h.** Correlation between species fractions measured in the set of redundant networks containing GC.

a

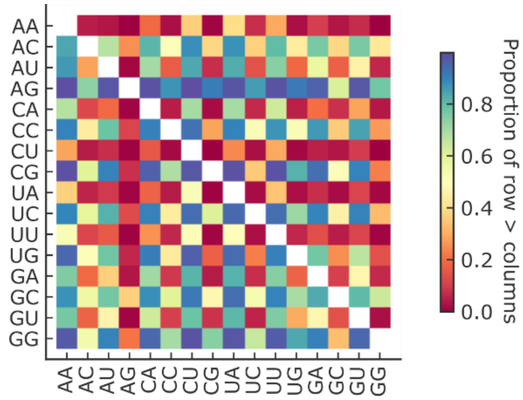

b

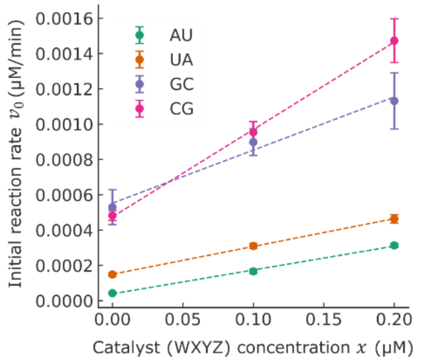

c

$$v_0 = \alpha x + \beta$$

Interaction $M \to N$	α(min^{-1})	β(μM.min^{-1})	r^2
$C \to G$	0.004950 ± 0.000283	0.000474 ± 0.000006	0.9993
$G \to C$	0.003008 ± 0.000335	0.000552 ± 0.000051	0.9833
$U \to A$	0.001575 ± 0.000097	0.000150 ± 0.000005	0.9998
$A \to U$	0.001352 ± 0.000041	0.000038 ± 0.000004	0.9975
$G \to U$	0.000160 ± 0.000007	0.000015 ± 0.000000	0.9997
$U \to G$	0.000066 ± 0.000001	0.000004 ± 0.000000	0.9986
$A \to G$	0.000018 ± 0.000000	0.000000 ± 0.000000	0.9279

e

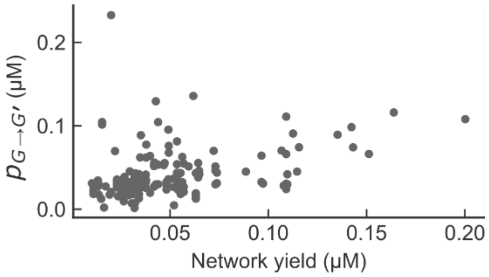

d

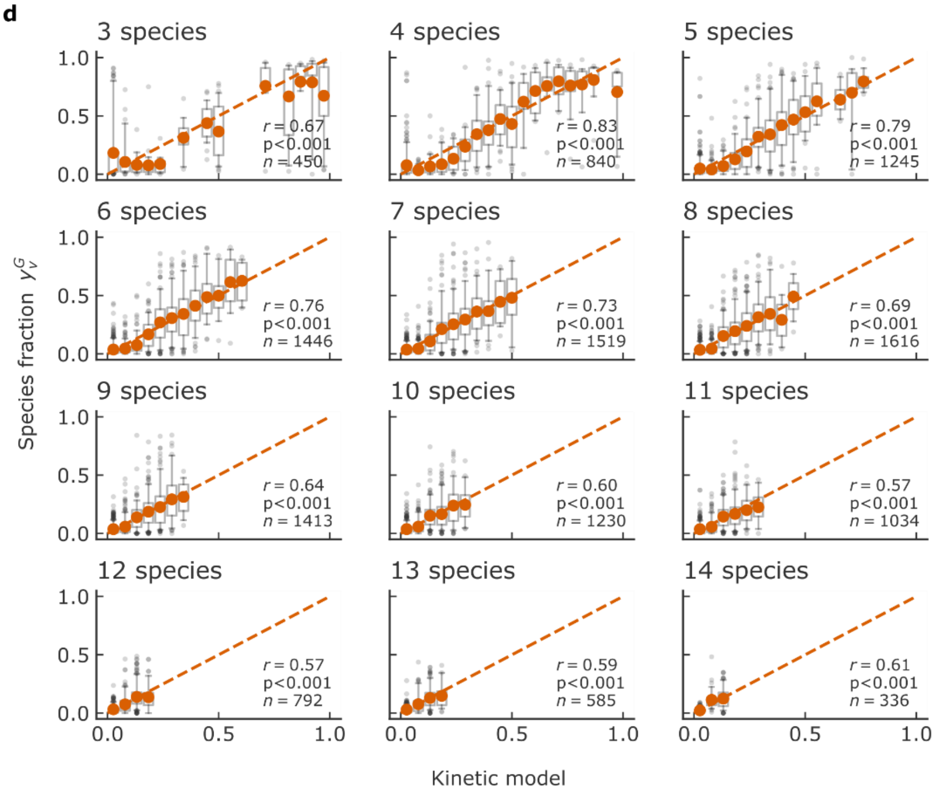

Supplementary Figure 6. Fitting of edge weights in *Azoarcus* networks across various models of species centrality and comparison of the best model with experimental measurements. a.

For each pair of species, fractions are extracted in all networks that contain the two species. The color code indicates the proportion of cases where one species (row) has higher fraction compared to the other species (column). **b-c** Experimental measurements to extract catalytic parameters used to describe the *Azoarcus* network system [2,3] (Methods) (Data are presented as mean values ±1 s.d over n=3 measurements per datapoint). **b.** Plot showing reaction kinetics for all four Watson-Crick IGS/tag interactions. Initial rates (v_0) of WXYZ formation from WXY and Z RNA fragments were measured by adding at the beginning of the reaction ('doping') covalent WXYZ ribozyme with the same IGS and tag as the WXY fragment, as reported by Yeates *et al.*[3]. **c.** Table showing the α and β parameters derived from fitting an affine relationship (top) between the initial rate of formation of covalent WXYZ ribozymes (v_0, measured as the initial slope of covalent ribozymes concentration over time) as a function of doped covalent WXYZ ribozyme concentration (x) [2,3]. Here α quantifies the synthesis of covalent ribozymes catalyzed by covalent ribozymes and, is derived from the slope of this relationship (plot in **b**). Whereas, β quantifies the synthesis of WXYZ ribozymes by non-covalent ribozymes and is derived from the intercept of this relationship (plot in **b**). **d.** Fraction obtained with the kinetic model versus measured fraction for networks with same number of species. Bins with fewer than 10 points were discarded. Dark grey dots are averages; quartile boxplots have 5th - 95th percentiles whiskers with flier points. The dotted orange line is the identity line. For each plot, n indicates the number of species and Pearson's correlation coefficient and p-value is reported (all p-values are <1e-5). **e.** Perturbation, computed with absolute concentrations in µM rather than species fraction as in Fig. 2d, for networks with 3, 4 and 5 species is plotted against network yield in µM for perturbations involving the addition of a novel catalysts (with G/C as the middle nucleotide of the IGS) with at least one target.

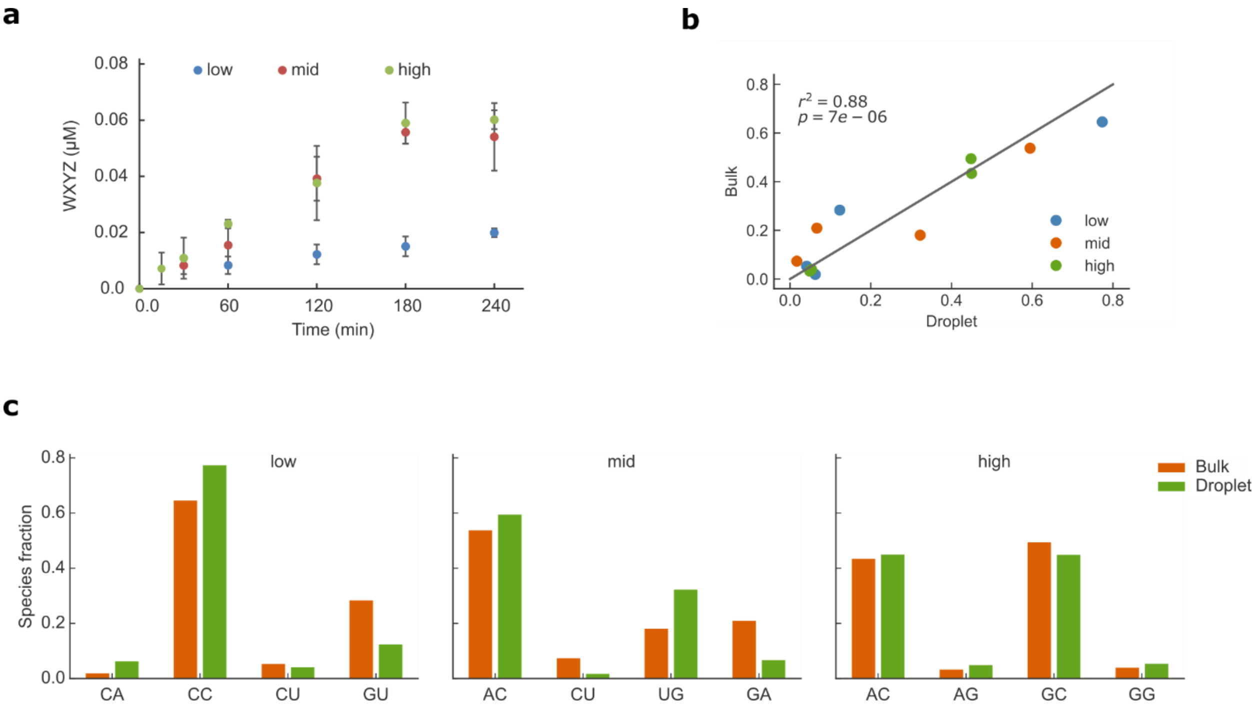

Supplementary Figure 7. Growth and composition measurement of representative examples of networks analysed by gel-electrophoresis and bulk-sequencing. a. Time-courses showing the amount of WXYZ catalyst formed in bulk for low-yield (CA, CC, CU, GU), mid-yield (AC, CU, UG, GA), and high-yield (AC, AG, GC, GG) 4 species networks, identified from droplet sequencing data (Fig. 2a), measured by gel-electrophoresis. RNA and $MgCl_2$ concentrations were identical to droplet experiments (see Methods for the details). Each measurement was performed in triplicate and mean with standard deviation plotted. **b.** Comparison of species fractions obtained from the networks (after 1 h incubation) using bulk sequencing with measured species fractions of the same networks observed in the droplet experiment dataset (Fig. 1d). Each dot represents the fraction of a species observed in bulk vs droplet sequencing experiment. **c.** The same networks as **a** and **b** but showing the species fraction of the individual species (WXYZ) in the network for all the three cases.

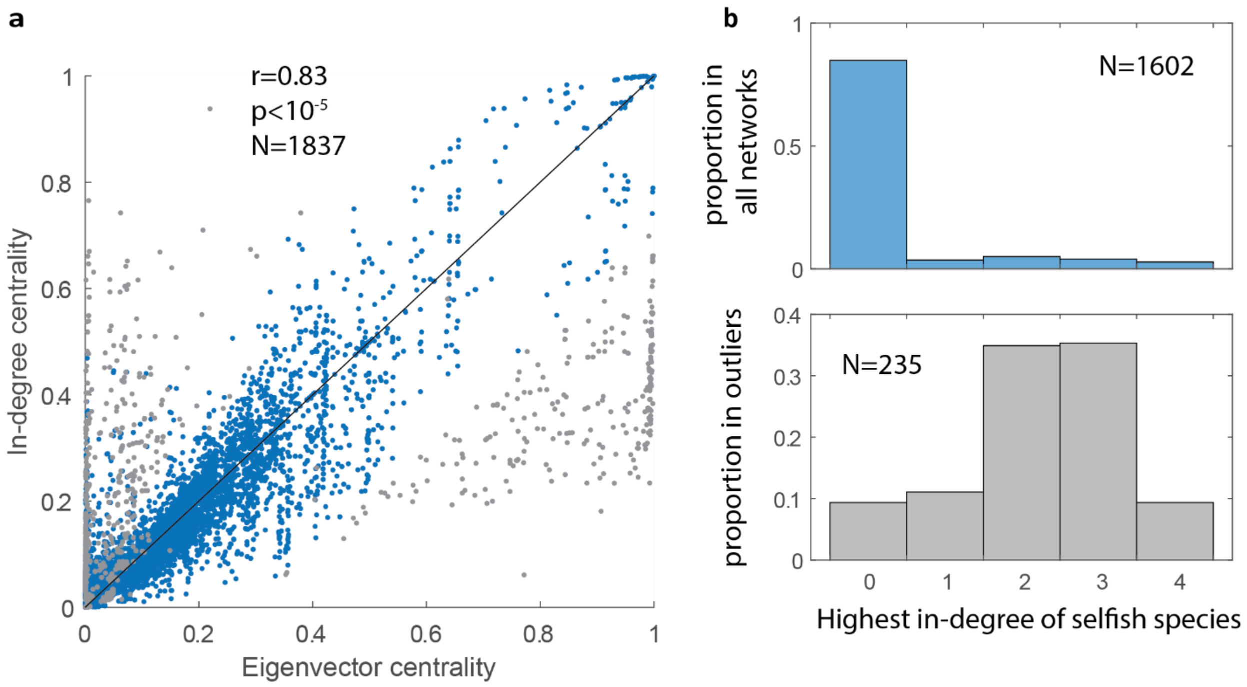

Supplementary Figure 8. Network composition predictions using eigenvector centrality and in-degree centrality. a. Covalent ribozyme fraction predicted by eigenvector centrality versus in-degree centrality for all networks of the experimental dataset (N=1837). In grey are species participating to networks containing outlier ribozymes. The latter are ribozyme species which fraction is underestimated by more than 20% by the in-degree centrality compared to the eigenvector centrality. Pearson's correlation coefficient and associated p-value is reported. **b.** Networks containing outliers tend to have selfish species (bottom) compared to others (top). Selfishness is quantified here as the in-degree of species with self-loops and no out-going edges.

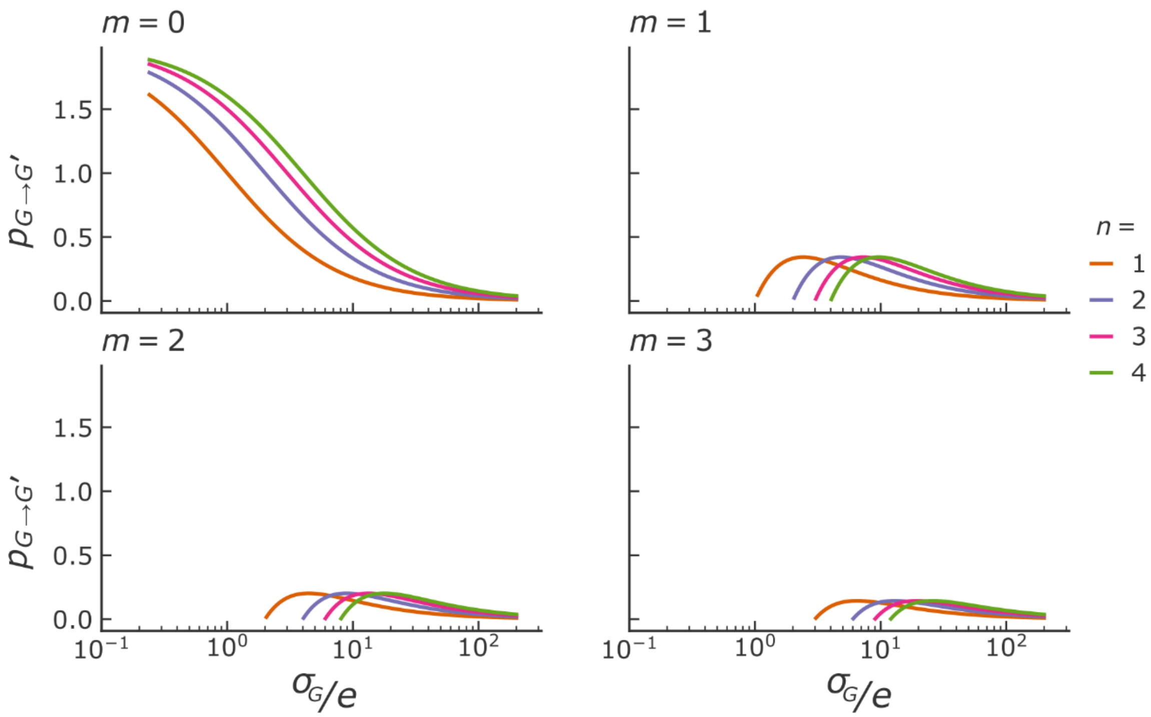

Supplementary Figure 9. Analytical predictions of Equation 1 with all possible values of the parameters n and m. Network perturbation $p_{G\to G'}$ upon addition of a new species is plotted against normalized background strength σ_G/e for all possible values of the catalytic novelty m (panels) and of perturbation breadth n (color coded).

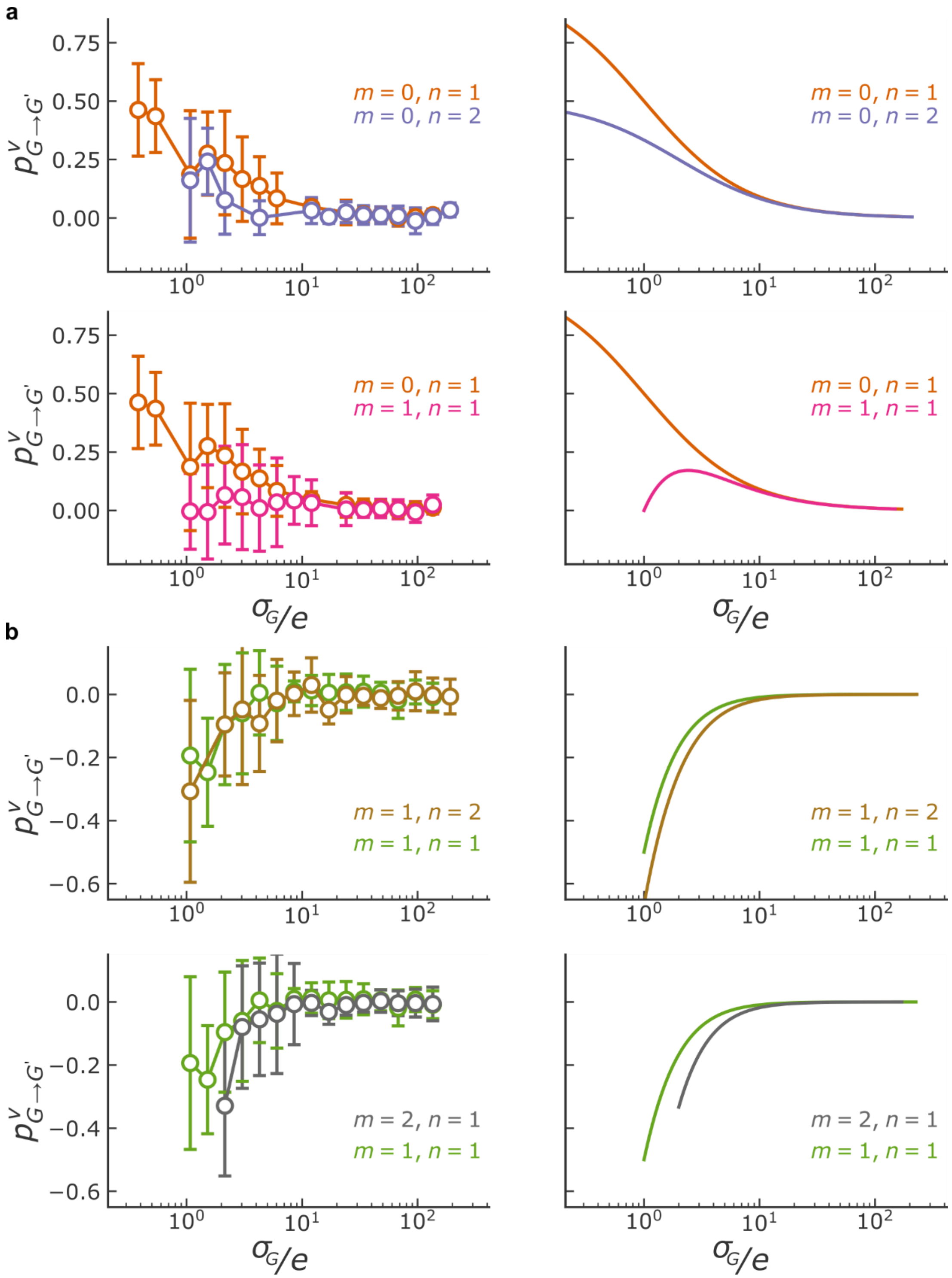

Supplementary Figure 10. Species perturbation $p^v_{G\to G'}$ upon addition of a new species a to network G, comparing experimental data with analytical predictions. a. $p^v_{G\to G'}$ plotted against

normalized background strength σ_G/e for different values of catalytic novelty m and perturbation breadth n in the case where v is a target for a and for both the experimental data (left) and the analytical results (right). For details of how analytical predictions were derived see Supplementary Notes. Data points were distributed along the x-axis in 20 log-spaced bins between $10^{-0.5}$ and $10^{2.5}$. Bins with fewer than 3 points were discarded and the mean and standard deviation for each bin are plotted. Networks with less than 3 species were discarded for the analysis. **b.** Same as **a** but the case where v is a not target for a.

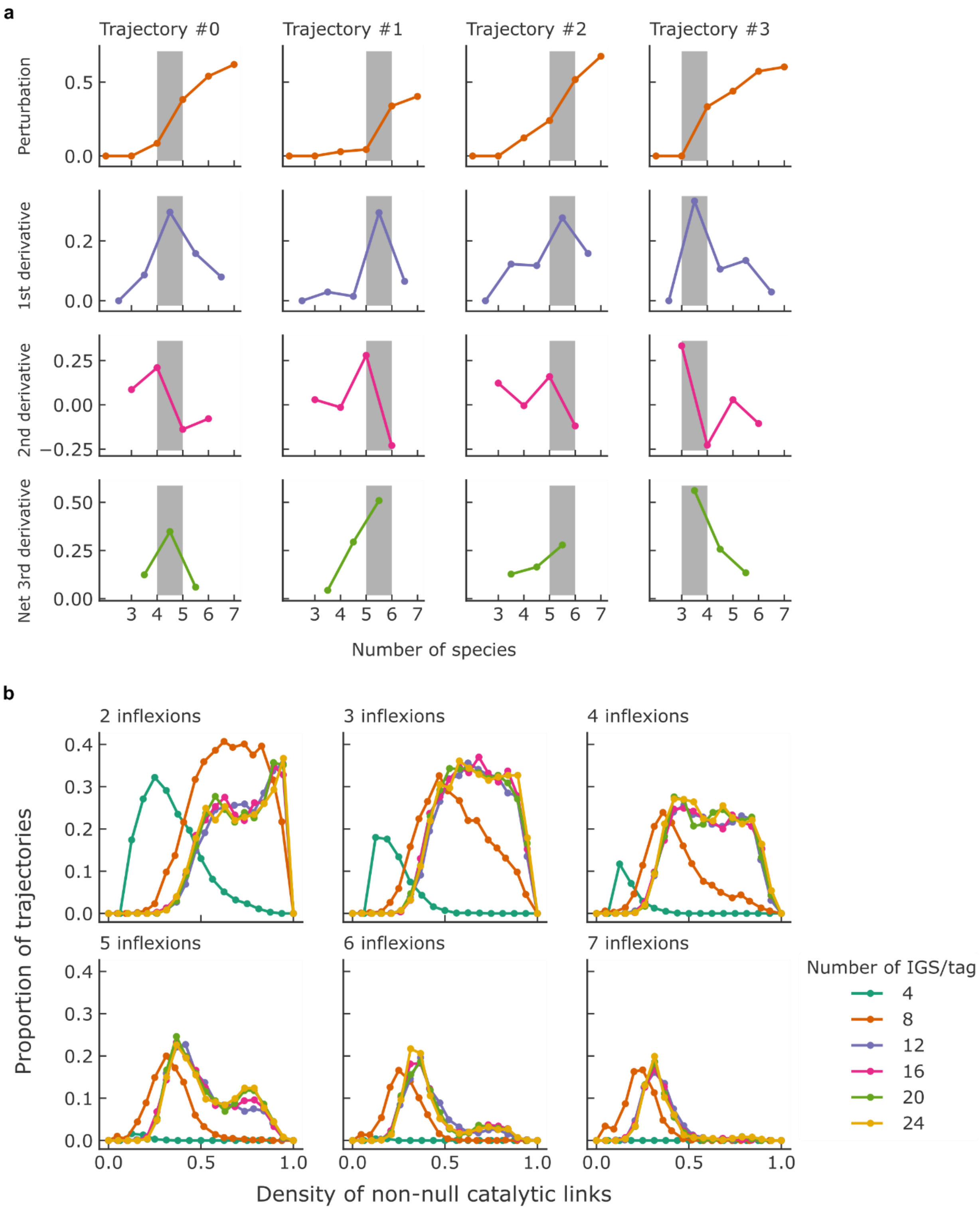
a
Trajectory #0
Trajectory #1
Trajectory #2
Trajectory #3
Perturbation
1st derivative
2nd derivative
Net 3rd derivative
Number of species
b
2 inflexions
3 inflexions
4 inflexions
5 inflexions
6 inflexions
7 inflexions
Proportion of trajectories
Density of non-null catalytic links
Number of IGS/tag
4
8
12
16
20
24

Supplementary Figure 11. Determination of strong inflexion points and influence of the number of different IGS/tag interactions on the number of inflexion points per trajectory. a. Cumulative perturbation for four representative trajectories from the experimental data are plotted (first row) as well as the first derivative (second row), the second derivative (third row) and the net third derivative (fourth row). The derivative is the difference between two successive points divided by the width of the interval (here 1). Inflexion points are first detected with the second derivative when it switches from positive to negative and the sharpness of these is measured with the net third derivative. Derivatives were computed as the difference of values between n and n+1 species. Strong inflexion points are defined as being in the top 25% in sharpness and are indicated with dark grey rectangles. An extra-step with no perturbation is added at the beginning of the trajectory to detect starting strong inflexion points. **b.** Proportion of trajectories with a given number of inflexions plotted against catalytic density for different numbers of IGS/tag combinations. Note that panels corresponding to 6 and 7 inflexions show no trajectory with inflexions when the number of IGS/tag pairs equals 4. Hence analysis of trends holds only when the number of IGS/tag pairs exceeds the number of inflexions, as mentioned in the main text. These results were obtained by simulating 20,000 trajectories as a function of catalytic density values (defined as the proportion of the non-null specific interaction between an IGS and a tag over the complete set of possible interactions) for various number of IGS/tag. See Fig. 4g for the details.

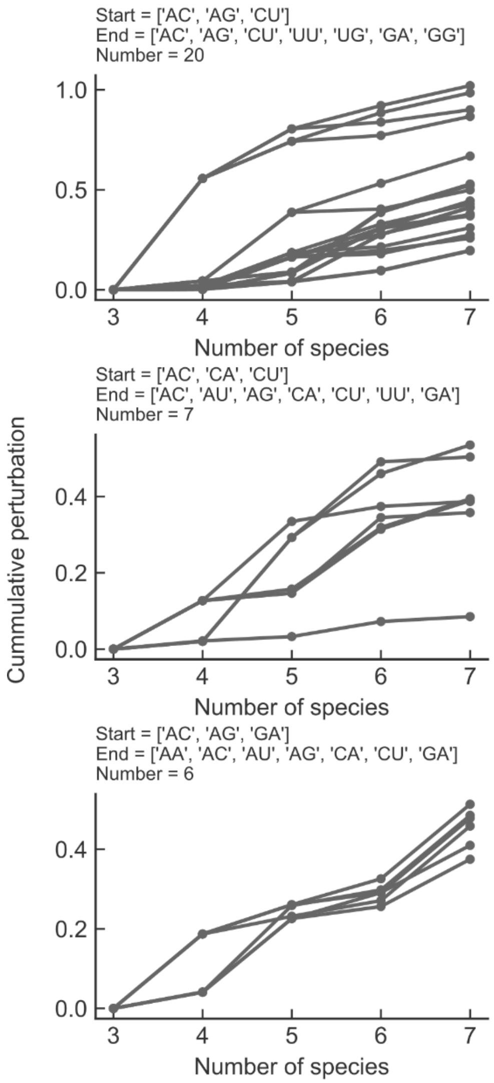

Supplementary Figure 12. Examples of alternative trajectories starting and ending with the same networks. Cumulative perturbation over trajectories is plotted against the number of species additions. Starting and ending networks correspond to the three trajectories of the middle panel of Fig. 4b.

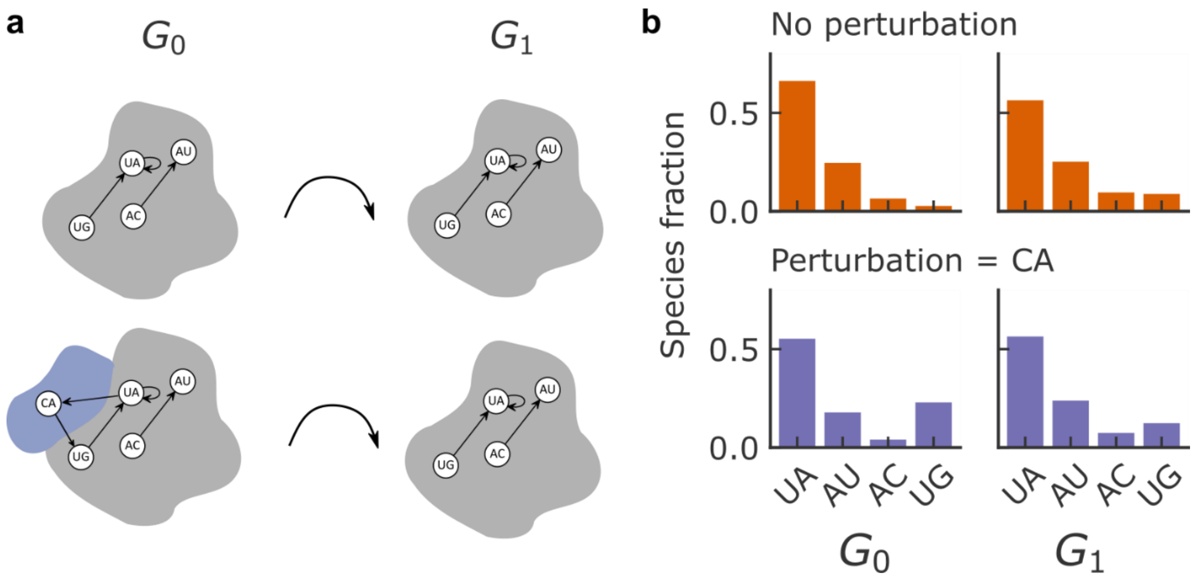

Supplementary Figure 13. Relaxation of chemical compositions to states due to non-covalent catalysts. a. Schematic of two serial transfer experiments with different initial conditions and two generations, G_0 and G_1, incubated for an hour at 48°C as in the droplet experiment. Top: control experiment (unperturbed network), where only the fragment for the AC, AU, UA and UG ribozymes are provided at each round, with concentrations 0.1 μM for WXY fragments and 1.6 μM for Z. Bottom: same as top, but the network is perturbed at G_0 by addition of CA fragments at the same concentration. **b.** Relative fraction of covalent ribozymes AC, AU, UA and UG produced during each generation of the serial transfer. Although introduction of fragments at G_0 has a significant impact on the final covalent ribozyme composition, these differences are not preserved at G_1 where the composition relaxes toward the one of the unperturbed networks.

Supplementary Table 1. Different combination of WXY fragments in initial 5 pL emulsions.

Combination id.	$_{gMg}$WXY$_{cNu}$ fragments (MN)[§]	Hairpin RNA reporter variable region[#]	Combinations that can be obtained from other combinations
1	CA	ACUG	
2	GA	AAUU	
3	AG	GAUA	
4	CU	GUGG	
6	UU	CCCA	
8	AC	GGAA	
15	GG	GAAU	
23	UC	AUGC	
18	GU	UUGG	
11	AU	UGUA	
0	UA	UUUU	
5	GC	UAUG	
17	GC	CGCC	
13	AC, AG	CGUG	3+8
21	CU, UU	AAAA	4+6
22	CA, CU	CUAA	1+4
7	CU, UG	AAGG	
10	GC, GG	UAAC	5+15, 15+17
19	CA, CC, GU	GUCC	
20	CC, GU, GG	UAGC	
16	AA, AU, AG, CA	GGGU	
12	CA, CC, UC, GA	UACA	
9	AU, CA, CG, UA, GA	AUAG	
14	AG, CG, UA, UC, UU, UG, GU, GG	GCCG	

[§]The initial combinations were generated randomly under constraints, including the coverage of diverse network sizes, as reported in the Methods (section 'Initial combinations of WXY fragments'). $_{gMg}$WXY$_{cNu}$ denotes RNA fragments where 'M' is the middle nucleotide of IGS and 'N' is the middle nucleotide of tag. A table of the catalytic rates between species is given in Supplementary Fig. 6c.

[#]Here only the variable region of the hairpin RNA reporter added to barcode the composition is reported. For the complete sequence please refer to Supplementary Data 1.

Supplementary Table 2. Variation in yield for different sizes of networks.

Number of species	Number of networks§	Mean yield (nM)	SD yield (nM)	Minimum yield (nM)	Maximum yield (nM)	Fold difference max/min yield	p-value#
2	22	37.3	19.1	10.4	95.1	9.1	2.3e-05
3	48	49.6	29.5	19.3	151.2	7.8	7.3e-09
4	60	56.0	26.4	25.6	146.9	5.8	2.7e-15
5	47	84.0	37.5	26.4	162.1	6.1	1.2e-05
6	47	113.9	43.8	52.4	233.3	4.5	1.6e-05
7	27	142.7	38.6	91.9	222.6	2.4	2.8e-08
8	17	181.7	67.9	92.6	373.1	4.0	1.3e-07
9	9	280.6	45.4	184.4	328.3	1.8	0.033
10	16	303.4	60.3	203.4	405.5	2.0	0.0092
11	10	371.9	86.9	236.8	481.3	2.0	0.0018
12	15	371.3	95.7	240.8	654.6	2.7	0.03
13	10	367.8	70.7	260.2	452.6	1.7	0.027
14	5	419.1	36.2	381.0	459.5	1.2	0.25
15	3	394.9	40.7	359.8	439.5	1.2	0.16

§Networks with less than 10 replicates were discarded for this analysis.
#We report the p-value from a one-tailed Student's t-test comparing mean yields between the pair of networks with minimum and maximum yield for each network size.

Supplementary References

1 Vaidya, N. *et al.* Spontaneous network formation among cooperative RNA replicators. *Nature* **491**, 72-77, (2012).

2 von Kiedrowski, G. A Self-Replicating Hexadeoxynucleotide. *Angew. Chem. Int. Ed. Engl.* **25**, 932, (1986).

3 Yeates, J. A. M., Hilbe, C., Zwick, M., Nowak, M. A. & Lehman, N. Dynamics of prebiotic RNA reproduction illuminated by chemical game theory. *Proc. Natl. Acad. Sci. U.S.A.* **113**, 5030-5035, (2016).
