# Supplementary material for: Darwinian properties and their trade-offs in autocatalytic RNA reaction networks
Source: Nat Commun. 2021 Feb 8;12:842. doi: 10.1038/s41467-021-21000-1 (PMC7870898; doi:10.1038/s41467-021-21000-1)
Supplement: Supplementary file 2 — Description of Additional Supplementary Files [file 41467_2021_21000_MOESM2_ESM.pdf]

Description of Additional Supplementary Files

Supplementary Movie 1.

Movie file showing the combinatorial electrocoalescence between 5 pL and 50 pL droplets. See Methods for the details.

Supplementary Movie 2.

Movie showing the droplet barcoding where deformable barcoded hydrogel beads are singly encapsulated in droplets and later fused with RNA droplet on the same device (not shown here). See Methods for the details.

Supplementary Data 1.

This file contains sequences of all oligonucleotides used in the study including primer, droplet barcode indexes, and multiplexing primer sequences. Additionally, it also contains parameters for the barcode indexes design.

Supplementary Data 2.

This file contains composition (relative fraction of each) of all the networks obtained from the experimental set-up as well as from kinetic model.

Supplementary Data 3.

AutoCAD file contain designs of all the microfluidic chips used in the study.
