# Supplementary material for: Darwinian properties and their trade-offs in autocatalytic RNA reaction networks
Source: Nat Commun. 2021 Feb 8;12:842. doi: 10.1038/s41467-021-21000-1 (PMC7870898; doi:10.1038/s41467-021-21000-1)
Supplement: Supplementary file 8 — Reporting Summary [file 41467_2021_21000_MOESM8_ESM.pdf]

nature research

Corresponding author(s): Philippe Nghe, Andrew D. Griffiths

Last updated by author(s): Dec 15, 2020

Reporting Summary

Nature Research wishes to improve the reproducibility of the work that we publish. This form provides structure for consistency and transparency in reporting. For further information on Nature Research policies, see our Editorial Policies and the Editorial Policy Checklist.

Statistics

For all statistical analyses, confirm that the following items are present in the figure legend, table legend, main text, or Methods section.

n/a	Confirmed	
☐	☒	The exact sample size (n) for each experimental group/condition, given as a discrete number and unit of measurement
☐	☒	A statement on whether measurements were taken from distinct samples or whether the same sample was measured repeatedly
☐	☒	The statistical test(s) used AND whether they are one- or two-sided<br>*Only common tests should be described solely by name; describe more complex techniques in the Methods section.*
☒	☐	A description of all covariates tested
☐	☒	A description of any assumptions or corrections, such as tests of normality and adjustment for multiple comparisons
☐	☒	A full description of the statistical parameters including central tendency (e.g. means) or other basic estimates (e.g. regression coefficient) AND variation (e.g. standard deviation) or associated estimates of uncertainty (e.g. confidence intervals)
☐	☒	For null hypothesis testing, the test statistic (e.g. F, t, r) with confidence intervals, effect sizes, degrees of freedom and P value noted<br>*Give P values as exact values whenever suitable.*
☒	☐	For Bayesian analysis, information on the choice of priors and Markov chain Monte Carlo settings
☒	☐	For hierarchical and complex designs, identification of the appropriate level for tests and full reporting of outcomes
☐	☒	Estimates of effect sizes (e.g. Cohen's d, Pearson's r), indicating how they were calculated

Our web collection on statistics for biologists contains articles on many of the points above.

Software and code

Policy information about availability of computer code

Data collection	Sequencing data was obtained processed (in the form of fastq files) from the sequencing platform (Genotyping and Sequencing Core Facility, ICM Paris). For extract data from gels, ImageJ (v1.49, v1.52) was used. For designing microfluidic chips, AutoCAD (2014, Student version) was used.
Data analysis	Custom code for processing sequencing data and for data analysis was written Python 2.7.16 and is available at https://doi.org/10.5281/zenodo.4016905. The compositional landscape (Fig. 1d) was prepared used 'Circos' software (Version 0.69-6).

For manuscripts utilizing custom algorithms or software that are central to the research but not yet described in published literature, software must be made available to editors and reviewers. We strongly encourage code deposition in a community repository (e.g. GitHub). See the Nature Research guidelines for submitting code & software for further information.

Data

Policy information about availability of data

All manuscripts must include a data availability statement. This statement should provide the following information, where applicable:

- Accession codes, unique identifiers, or web links for publicly available datasets
- A list of figures that have associated raw data
- A description of any restrictions on data availability

The dataset of species fractions and number of UMIs for all networks is provided as Supplementary Data 2. Raw datasets are available at https://doi.org/10.6084/m9.figshare.c.5103962.

Field-specific reporting

Please select the one below that is the best fit for your research. If you are not sure, read the appropriate sections before making your selection.

- [x] Life sciences
- [ ] Behavioural & social sciences
- [ ] Ecological, evolutionary & environmental sciences

For a reference copy of the document with all sections, see nature.com/documents/nr-reporting-summary-flat.pdf

Life sciences study design

All studies must disclose on these points even when the disclosure is negative.

Sample size	The high-throughput nature of the method ensures a high number of samples (>20,000 droplets, >1,800 unique networks).
Data exclusions	During sequencing data processing, ~30% of the reads were discarded because it was not possible to identify one of the meta fields that are be associated with each read (Droplet barcode, UMI, IGS, tag and hairpin RNA reporter). The reads with the same meta information were merged and the read count was added as an extra meta field. UMI-normalized with low read count were considered as noise and discarded (Supplementary Fig 3e-f). During data analysis, filtered UMI-normalized reads were then clustered by droplet barcode. Droplet with less than 10 UMI-normalized hairpin reads and less than 20 UMI-normalized ribozyme reads were discarded. This is described in greater length in the Methods under section `Sequencing data processing`.
Replication	Replicability was tested with a full experimental replicate (Supplementary Fig. 5c).
Randomization	Randomization is not applicable since there are no group allocation in this study.
Blinding	Blinding is not applicable since there are no group allocation in this study.

Reporting for specific materials, systems and methods

We require information from authors about some types of materials, experimental systems and methods used in many studies. Here, indicate whether each material, system or method listed is relevant to your study. If you are not sure if a list item applies to your research, read the appropriate section before selecting a response.

Materials & experimental systems

n/a	Involved in the study	
x		Antibodies
x		Eukaryotic cell lines
x		Palaeontology and archaeology
x		Animals and other organisms
x		Human research participants
x		Clinical data
x		Dual use research of concern

Methods

n/a	Involved in the study	
x		ChIP-seq
x		Flow cytometry
x		MRI-based neuroimaging

April 2020
